# Controllability of complex networks with unilateral inputs

**DOI:** 10.1038/s41598-017-01846-6

**Published:** 2017-05-12

**Authors:** Gustav Lindmark, Claudio Altafini

**Affiliations:** Division of Automatic Control, Dept. of Electrical Engineering, Linköping University, SE-58183 Linköping, Sweden

## Abstract

In this paper, we study the problem of controlling complex networks with unilateral controls, i.e., controls which can assume only positive or negative values, not both. Given a complex network represented by the adjacency matrix A, an algorithm is developed that constructs an input matrix B such that the resulting system (A, B) is controllable with a near minimal number of unilateral control inputs. This is made possible by a reformulation of classical conditions for controllability that casts the minimal unilateral input selection problem into well known optimization problems. We identify network properties that make unilateral controllability relatively easy to achieve as compared to unrestricted controllability. The analysis of the network topology for instance allows us to establish theoretical bounds on the minimal number of controls required. For various categories of random networks as well as for a number of real-world networks these lower bounds are often achieved by our heuristics.

## Introduction

In Engineering sciences, the concept of controllability has a long history, with a large body of theory and many ramifications covering different aspects of the problem^1, 2^. In recent times, the topic has received a considerable attention in connection with the study of complex networks^3–9^. Complex networks appear in a broad spectrum of scientific disciplines, ranging from Biology to Social Sciences, from Technology to Engineering. Controlling a complex network means steering the state variables associated with its nodes to an arbitrary state using the available control inputs. When a network is given but there is no a-priori information on how it can be controlled, then an interesting problem is to find a minimal set of driver nodes (i.e. nodes on which an external control input is acting) that render the network controllable^4, 5^. This problem has elegant solutions when linear dynamics is assumed and the amplitude of the control inputs is unrestricted. Different approaches are discussed in the literature. The simplest situation is when both topology and interaction strength of the connections are completely known (i.e. the weighted adjacency matrix of the network is available). In this case standard controllability tests such as the Kalman rank condition^10^ or the PBH test can be used^7, 11, 12^. Another situation that has received a lot of attention is when only the topology of a network is available but not the exact weights. Concepts like structural controllability (i.e. a controllability notion that holds for almost all values of the edge weights) have then been used^4–6^.

All the publications mentioned so far present general controllability results based on the assumption that the control inputs are unrestricted i.e. they can assume any value. However in many different fields in which controllability of large scale networks is studied, the control action is intrinsically constrained. In the literature this has been considered for some specific network control problems^13^. The most common form of constraint is that control inputs are *unilateral*, i.e. they can assume either positive or negative values, but not both. For instance, in a biological network where nodes correspond to molecular components, a drug acting on a molecule can be considered a control input. Usually its mode of action is to either activate its target or to inhibit it, not both. Complex networks in which inputs are naturally unilateral occur in many other domains, such as for instance in transportation, in trade, or in power networks, see Table 1 for more motivating examples.

**Table 1:** Application areas with naturally unilateral control inputs.

Network type	Unilateral controls
Biology	In a network with nodes corresponding to molecular components, a drug acting on a molecule can be considered a control input, and acts by activating or inhibiting its target, not both.
Power grid	In controlling the power flow over a power network, loads are absorbing power but not producing it, while generators normally play the opposite role.
Transport	Measures to reduce the capacity/flow through a node in a road network are for instance traffic lights or variable speed limitations or tolls. For an air transport network, increasing/decreasing the number of flights at a node for instance through subsidies/fees can increase the airport capacity. Increasing/decreasing the number of departures towards a specific destination allows instead to modulate the flow over the edges.
Internet	Server workload can be controlled by redirecting or declining tasks. Different processes or clients may be allocated bandwidth dynamically based on priority.
Food-web	Food-webs describe how the species in an ecosystem interact. A species can be added by stocking/feeding or selectively reduced by hunting/fishing. Such measures can be regarded as unilateral control inputs.
Trade	Economic policies such as import restrictions and tolls as well as subsidies of the domestic production can be considered as unilateral controls in international trade networks.
Water distribution	Nodes represent junctions of pipes, reservoirs or tanks that are connected by pipes. For instance pumps and valves can take the role of unilateral controls.

For each of the classes of networks listed in the table, examples of constrained controls are provided. Most often these constraints take the form of unilateral inputs.

Also controllability with constrained inputs has a long history, see Chapter 5 of Jacobson^14^ for a survey focusing on driven control systems. A key result in this literature was obtained in Brammer^15^ based on the knowledge of the system eigenvalues. In this paper the classical results from Brammer^15^ are reformulated for unilateral controls and arbitrarily large networks. In order to do that, Brammer’s conditions for controllability are expressed using the theory of positively spanning sets^16, 17^. The problem of finding a minimal set of unilateral control inputs that guarantee controllability can then be formulated in terms of standard optimization problems. An algorithm is developed that constructs a near-minimal set of unilateral control inputs for a given network with linear dynamics. The performance of the algorithm is verified by comparison with theoretical bounds on the number of unilateral controls obtained from the network topology. Using the algorithm we study how the minimal number of unilateral controls, *N* ^*r*^, relates to the minimal number of unrestricted controls, *N* ^*u*^. We compare *N* ^*r*^ with *N* ^*u*^ for various categories of networks in order to find out what is the additional cost of adding sign constraints in a network controllability problem.

## Methods

### The minimal unilateral controllability problem

The networks that we study can be described by the continuous linear control system

1 $$\dot{x}(t)=Ax(t)+Bu(t),$$

where $A\in {{\mathbb{R}}}^{n\times n}$ and $B\in {{\mathbb{R}}}^{n\times m}$ are matrices, usually sparse. The vector $x(t)\in {{\mathbb{R}}}^{n}$ represents the state of the *n* “agents” (i.e. nodes) and *u*(*t*) is the vector of the *m* control inputs. The directed graph of the network, denoted ${\mathscr{G}}(A)$, is the set of nodes *ψ*_1_, …, *ψ*_*n*_ and edges (*ψ*_*i*_, *ψ*_*j*_), *i*, *j* s.t. *A*_*ji*_ ≠ 0, with weights given by the numerical values of the elements in *A*.

Only control inputs that act on a single node (driver node) are considered in the following. This corresponds to assuming that the columns of *B* are elementary vectors, i.e., have only one non-zero entry. The problem of finding a minimal set of driver nodes that renders the system (1) controllable is studied in several recent publications^4–7^. In this literature the control inputs are assumed unrestricted, i.e., an input *u*_*i*_ can take values anywhere in ${\mathbb{R}}$. The minimum number of unrestricted control inputs that guarantee controllability of (1), *N* ^*u*^, can be interpreted as a measure of how difficult it is to control a certain network^4^.

Constraints on the control inputs can be formalized by introducing a *control restraint set*, ${\rm{\Omega }}\subset {{\mathbb{R}}}^{m}$. The admissible controls are then all vector functions *u*(*t*) taking value in the control restraint set,

2 $$u(t)\in {\rm{\Omega }},\forall t\mathrm{.}$$

In particular, when the control inputs are unilateral we have ${\rm{\Omega }}={{\mathbb{R}}}_{+}^{m}$, and the sign of the elements of *B* determine if the control action is positive or negative. Assume that we are free to add control inputs acting on any single node either positively or negatively. Then *B* has the following form:

3 $$B=[\begin{array}{c}\pm {e}_{{i}_{1}}\ldots \pm {e}_{{i}_{m}}\end{array}]$$

with *e*_*i*_ the *i*-th elementary vector, *i* ∈ 1, …, *n*. It is not a restriction to assume unit gain on the columns of *B* as this only corresponds to a rescaling of the inputs.

Our first task can then be formulated as follows: given ${\mathscr{G}}(A)$, determine the minimal number of unilateral control inputs *N* ^*r*^ so that the system (1) with the unilateral controls (2) is controllable.

### Conditions for unilateral controllability

Conditions for controllability of the linear system (1) subject to the control restraint (2) were derived in Brammer^15^. Brammer’s necessary and sufficient conditions for controllability are reported in the Supplemental Information (SI). The key points are that

(i) Rank [*B AB A* ^2^ *B* … *A* ^*n*−1^ *B*] = *n*;
(ii) There is no real left eigenvector *v* of *A* s.t. $\langle v,Bu\rangle \leqslant 0\forall u\in {\rm{\Omega }}$.

The first condition is the same as for controllability with unrestricted controls. The second condition is instead specific for systems with control restrains and describes how such inputs have to excite the real modes of the system.

When the control restraint set is ${\rm{\Omega }}={{\mathbb{R}}}_{+}^{m}$, i.e. the control inputs are unilateral, we can reformulate Brammer’s condition in more suitable terms using the theory of *positive linear dependence* ^16^. See the SI for an overview of this topic. Here, the concept of *positive span* is important: The vectors ${a}_{1},\ldots ,{a}_{r}\in {{\mathbb{R}}}^{n}$ positively span ${{\mathbb{R}}}^{n}$ if for any $x\in {{\mathbb{R}}}^{n}$, ∃ *θ*_1_, …, *θ*_*r*_ ≥ 0 s.t. *x* = *θ*_1_ *a*_1_ + … + *θ*_*r*_ *a*_*r*_. The number of vectors needed to accomplish this is bounded by *r* ≥ *n* + 1, a number which is achieved if for instance *a*_1_, …, *a*_*n*_ *linearly span* ${{\mathbb{R}}}^{n}$ and *a*_*n*+1_ = −(*a*_1_ + … + *a*_*n*_).

In order to investigate controllability with unilateral control inputs, the eigenspaces corresponding to the real eigenvalues of *A* must be determined. Since the eigenvalues of *A* depend on the numerical entries of *A*, the problem is not generic in the structural controllability sense^18, 19^. Assume ${\lambda }_{0}=0,{\lambda }_{1},\ldots ,{\lambda }_{\ell }$ are the real eigenvalues of *A* of geometric multiplicity ${\mu }_{0},{\mu }_{1},\ldots ,{\mu }_{\ell }$. Let ${V}_{i}=[{v}_{i\mathrm{,1}}\ldots {v}_{i,{\mu }_{i}}],i=\mathrm{0,1},\ldots ,\ell$, be a matrix whose columns form a basis for the left eigenspace associated with the real eigenvalue *λ*_*i*_. Then Brammer’s condition becomes:

**Theorem 1**. *The system* (1) *is controllable with unilateral control inputs if*

(i) *The matrix* [$B\,{AB}\,{A}^{2}$ *B … A* ^*n*−^ ^1^ *B*] *has rank n*.
(ii) *The columns of* ${V}_{i}^{T}B$ *positively span* ${{\mathbb{R}}}^{{\mu }_{i}}$ $\forall i=0,1,\ldots ,{\ell }$.

A proof of this theorem is provided in the SI. Condition (*i*) of the theorem will in the following be referred to as the rank condition and condition (*ii*) as the positive span condition. Since *u* ≥ 0, the set ${C}_{i}=\{{{\rm{V}}}_{i}^{T}Bu,u\ge 0\}$ is a cone, hence the positive span condition (*ii*) of the theorem can be rephrased as all cones ${{\mathscr{C}}}_{i}$, $i=0,\ldots ,\ell$, being *simultaneously* vector spaces of dimension equal to the geometric multiplicities ${\mu }_{0},\ldots ,{\mu }_{\ell }$ of the real eigenvalues ${\lambda }_{0},\ldots ,{\lambda }_{\ell }$ of *A*. In order to do that, it is enough that each ${{\mathscr{C}}}_{i}$ is not contained in any half space of ${{\mathbb{R}}}^{{\mu }_{i}}$, see Fig. S1 for an illustration.

### Constructing a minimal set of unilateral control inputs

**Figure 1:**
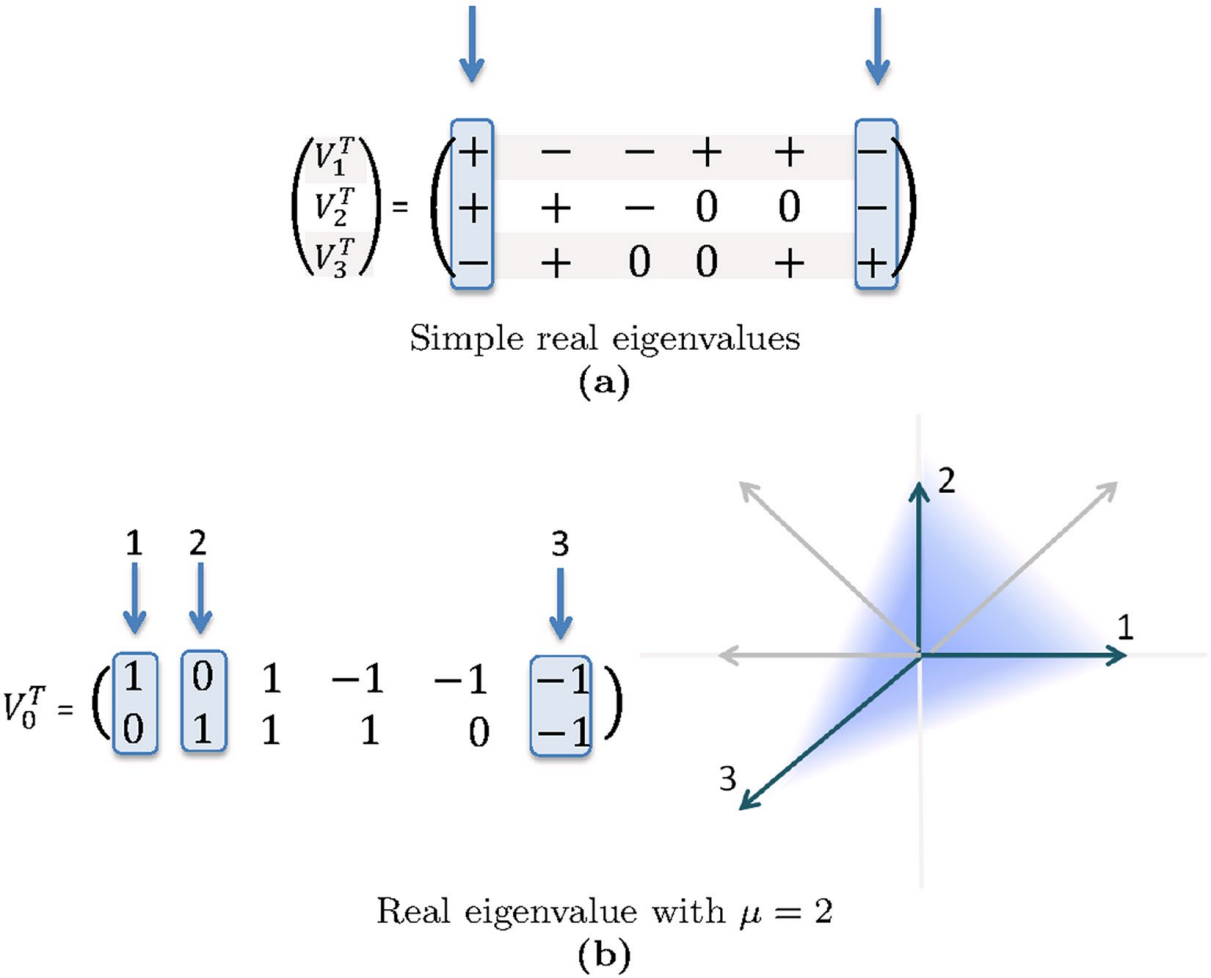
Constructing a minimal set of unilateral control inputs. (**a**) When the real eigenvalues of *A* are simple, condition (*ii*) of Theorem 1 leads to a set cover problem: Each control input “covers” the row-vectors ${V}_{i}^{T}$ either positively or negatively or not at all. A minimal set of control inputs should be constructed such that all vectors are covered both positively and negatively. The two highlighted columns form a set cover solution and correspond to $$B=[\begin{array}{cc}{e}_{1} & {e}_{6}\end{array}]$$. (**b**) Consider the eigenspace of a real eigenvalue of geometric multiplicity *μ* = 2. The controllability condition is then a problem of positively spanning sets. The three highlighted columns correspond to $$B=[\begin{array}{ccc}{e}_{1} & {e}_{2} & {e}_{6}\end{array}]$$ and they positively span ${{\mathbb{R}}}^{2}$.

Assume that we are given a large network specified by the system matrix *A* and that our problem is to find a minimal number of unilateral control inputs such that the resulting system is controllable. Using the controllability conditions of Theorem 1, this problem can be reformulated in terms of well studied optimization problems. When the real eigenvalues of *A* are all simple, the matrices ${V}_{i}^{T}B,i=0,\ldots ,\ell$ are row-vectors. The positive span condition is met when each of them have both a positive and a negative entry. Our problem can then be formulated as a variant of the well known combinatorial *Set-Cover Problem* ^20^, see Fig. 1(a) for an illustration. When on the other hand there is a real eigenvalue *λ*_*i*_ with geometric multiplicity *μ*_*i*_ > 1, then the problem can be mapped into the computation of a minimal positively spanning set^21^, illustrated in Fig. 1(b). Both these optimization problems are NP-complete, meaning that finding the exact global minimum becomes infeasible when the size of the system grows.

The greedy heuristics which we use in this paper are described in detail in the SI. They do not guarantee optimality, but produce an approximate solution with a near-minimal set of control inputs.

### Unrestricted vs. unilateral control inputs

In the following of this paper we analyze the minimal number of unilateral controls, *N* ^*r*^, and compare it with *N* ^*u*^ for different classes of networks. Readers can consult for instance Rugh^1^ for a thorough treatment of the rank condition (*i*) of Theorem 1 which applies also when the control inputs are unrestricted. Instead, we will mostly elaborate on the positive span condition (*ii*) and assume that condition (*i*) is met.

Trivial bounds on *N* ^*r*^ are *N* ^*u*^ ≤ *N* ^*r*^ ≤ 2*N* ^*u*^. The upper bound is obtained when each unrestricted control input is replaced with an equivalent set of one positive and one negative unilateral control input acting on the same node as the original input. On the other hand, clearly *N* ^*r*^ ≥ *N* ^*u*^ because a system that is not controllable with a certain set of unrestricted control inputs cannot become controllable if these are constrained. We use the quotient *η* = *N* ^*r*^/*N* ^*u*^ ∈ [1, 2] as a relative measure of the ability to control a network using unilateral controls instead of unrestricted controls. Clearly the cases worthwhile exploring are those for which *η* is significantly less than two. A trivial case in which the conditions for unilateral resp. unrestricted controllability coincide and the lower bound *N* ^*r*^ = *N* ^*u*^ is achieved is when all eigenvalues of *A* are complex conjugate. In general, however, *A* will have one or more real eigenvalues, hence the problem of controlling with unilateral inputs is a non-trivial one. One important subtask is to understand when such real eigenvalues are induced by the topology and when instead by the numerical values of the edge weights.

**Figure 2:**
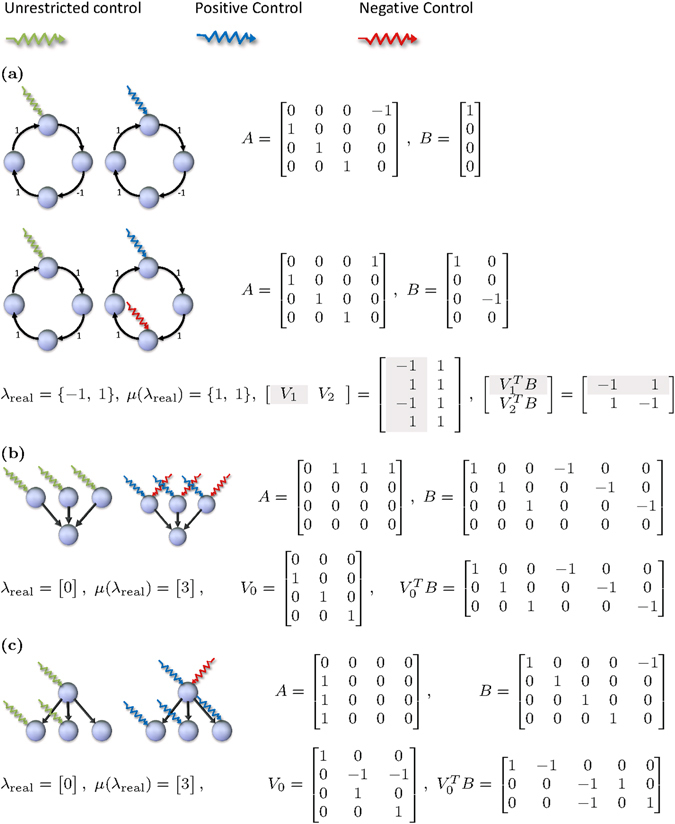
Examples of networks and control inputs that make them controllable. The system matrices *A* are shown with their real eigenvalues and left eigenvectors, as well as the minimal *B* that verifies condition (*ii*) of Theorem 1. (**a**) A circular network requires either one or two unilateral controls for controllability depending on the edge weights. When one edge weight is negative, all eigenvalues of *A* are complex and condition (*ii)* of Theorem 1 is trivially met. When all edge weights are positive, there are two real eigenvalues with eigenspaces of dimension 1. In this case condition (*ii*) of Theorem 1 is equivalent to requiring that each of the row vectors ${V}_{1}^{T}B$ and ${V}_{2}^{T}B$ have both a positive and a negative entry. (**b**) A hub with incoming edges. The only real eigenvalue is *λ*_0_ = 0 of geometric multiplicity 3. Three unrestricted or 6 unilateral controls are required for controllability. The columns of ${V}_{0}^{T}B$ positively span ${{\mathbb{R}}}^{3}$. (**c**) A hub with outgoing edges. 5 unilateral control inputs are enough to achieve controllability.

To illustrate what can happen, four simple networks are discussed in Fig. 2. Consider first the circular network in Fig. 2(a). All eigenvalues of *A* are complex when there is an odd number of edge weights with negative sign. In this case the positive span condition is trivially met and the rank condition is satisfied with one single control input, hence *N* ^*r*^ = *N* ^*u*^ = 1 and *η* = 1. When instead there is an even number of edge weights with negative sign, then at least one eigenvalue is real and the positive span condition comes into play. In this case *N* ^*r*^ = 2 and we conclude that *N* ^*r*^ in general depends on the numerical values of the edge weights of a network, see the SI for more details.

The zero eigenvalue must be handled separately in analyzing controllability of complex networks. We will use ${{\mathscr{N}}}_{\ell }(A)$ for the left null space of *A* and ${\mu }_{0}={\rm{\dim }}\,{{\mathscr{N}}}_{\ell }(A)$ for its dimension (which coincide with the geometric multiplicity). What is special with the zero eigenvalue is that its geometric multiplicity depends on topological properties of ${\mathscr{G}}(A)$. Any root node (i.e., a node having indegree *k*_in_ = 0) generates one eigenvalue. Also a “dilation” in the network, i.e. a set of nodes with a smaller set of in-neighbours (see the SI for formal definition), generates one or several zero eigenvalues. In the literature on structural controllability (where controls are unrestricted), *μ*_0_ is often referred to as the *rank deficiency* of the system^18, 22^. Control inputs (driver nodes) are required to target each root and dilation of ${\mathscr{G}}(A)$. This leads to the lower bound *N* ^*u*^ ≥ *μ*_0_ on the number of unrestricted controls^22^. Unlike other eigenvalues in for instance networks with random edge-weights (see below for more details), the geometric multiplicity of the zero eigenvalue, *μ*_0_, may be high. In the case of unilateral controllability, this results in a potentially very large problem of positive spanning sets.

Both the networks in Fig. 2(b,c) are acyclic and correspond to nilpotent adjacency matrices *A*, which means that *λ*_0_ = 0 is the only eigenvalue of *A* regardless of the numerical entries on the edges of ${\mathscr{G}}(A)$. In Fig. 2(b), a number of roots have outgoing edges to a hub, while in the network in Fig. 2(c), the directions of the edges are switched and we have a dilation. The number *N* ^*u*^ is the same for both networks, but *N* ^*r*^ differs: In Fig. 2(b), each root must be controlled with one unrestricted control input or two unilateral controls - one positive and one negative. However, the dilation in Fig. 2(c) requires less unilateral controls. In Table 2 this result is generalized to single layer trees with a single hub having arbitrary indegree and outdegree. In particular, in the limit *n* → ∞, *η* → 2 in the first case while *η* → 1 in the second case.

**Table 2:** The number of unrestricted or unilateral control inputs required for controllability of some specific network structures.

Network type	*N* ^*u*^	*N* ^*r*^	*η*
Circular network, positive cycle	1	2	2
Circular network, negative cycle	1	1	1
Hub with *n* − 1 outgoing edges	*n* − 1	*n* + 1	*η* → 1 when *n* → ∞
Hub with *n* − 1 incoming edges	*n* − 1	2(*n* − 1)	2
Rooted directed tree, outgoing edges from the root	*n*_leaf_	*n*_leaf_ + *r* + 1	(*n*_leaf_ + *r* + 1)/*n*_leaf_
Full network, simple eigenvalues	1	2	2
Full network, skew-symmetric	1	1	1

For the RDT, the number of leaves coincides with the dimension of the null space, i.e. *n*_leaf_ = *μ*_0_, and *r* is the number of nodes with outdegree ≥2.

### Rooted directed trees and directed acyclic graphs

The observations regarding indegree vs. outdegree can be extended to Rooted Directed Trees (RDTs) and Directed Acyclic Graphs (DAGs).

Also for them, *λ*_0_ = 0 is the only eigenvalue of *A* and only ${V}_{0}^{T}B$ must be considered when evaluating the positive span condition of Theorem 1 for unilateral controllability. For RDTs we can derive both *N* ^*r*^ and *N* ^*u*^ entirely from the topology: Expressions for *N* ^*r*^ and *N* ^*u*^ in terms of the number of nodes with outdegree ≥2 and leaves in the tree are stated in Table 2 and derived in the SI. In general, when the tree is “flat”, i.e. with few but broad layers, then *μ*_0_ and thus also *N* ^*u*^ are high, but the additional cost for using unilateral controls is low and *η* is close to 1. When instead the tree is deep (i.e. with many layers), then less unrestricted control inputs are required, but the ratio *η* approaches 2.

The same principles apply also to DAGs, although no explicit expressions for *N* ^*r*^ and *N* ^*u*^ are available. Controllability with unilateral control inputs is relatively easy to achieve for a DAG with one or a few roots and hubs with a majority of outgoing edges, as *η* is close to 1 in these cases. When instead hubs are indegree-dominated, then the graphs tend to have many roots, hence *N* ^*r*^ tends to be closer to its upper bound 2*N* ^*u*^ and *η* close to 2. In a DAG we can define the “depth”, *h*_max,*i*_, of node *ψ*_*i*_, *i* = 1, …, *n*, as the maximal path length from any root to *ψ*_*i*_. The depth of the whole DAG is defined as *h*_max_ = max_*i*=1,…,*n*_ *h*_max,*i*_. In general, a deep DAG requires few unrestricted or unilateral control inputs. In the extreme case where the DAG consists of a single path that runs from the root through all nodes, then *N* ^*u*^ = 1 and *N* ^*r*^ = 2.

**Figure 3:**
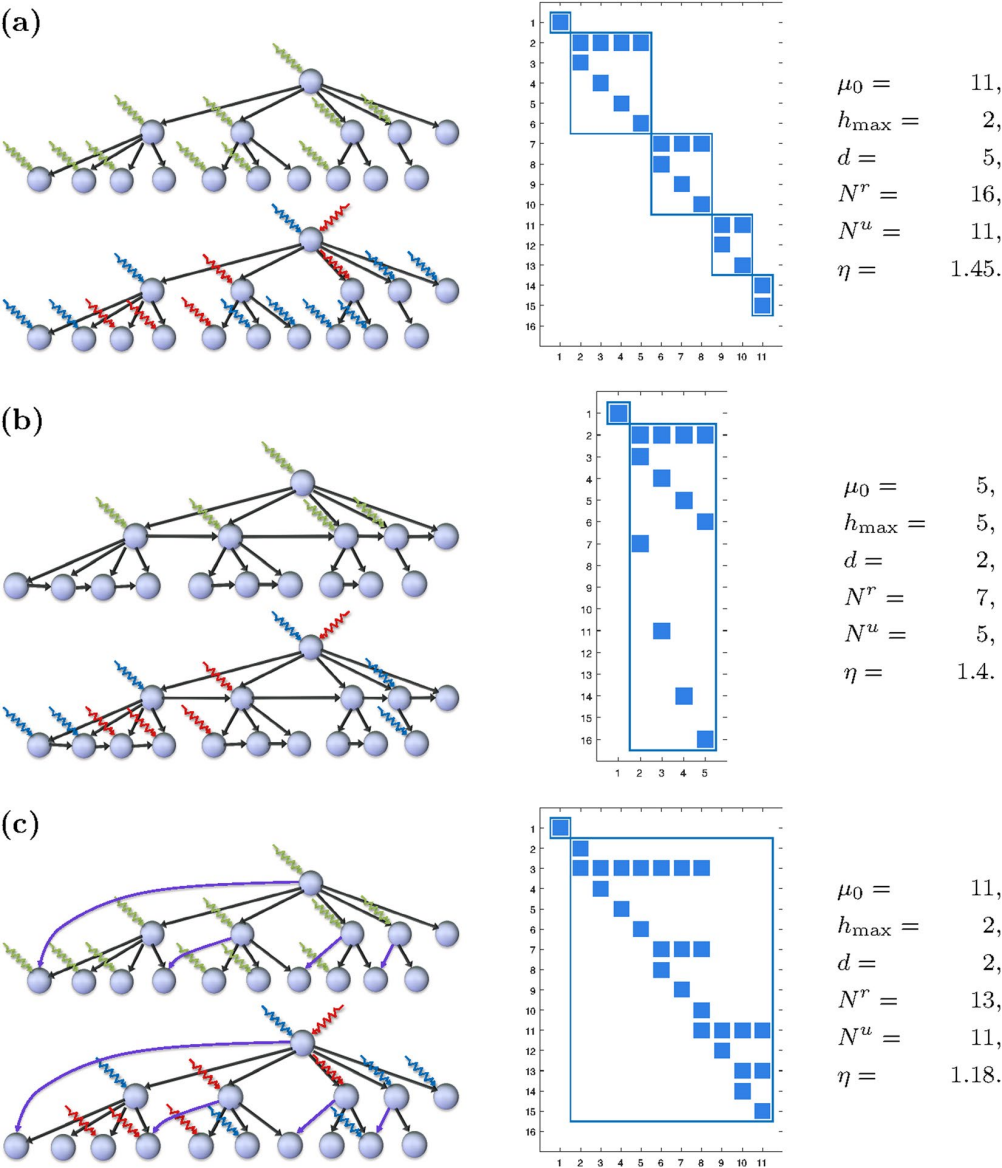
Rooted directed trees and directed acyclic graphs. (**a**) A RDT with 16 nodes of which 4 have outdegree ≥2 and 11 are leaves. For such tree it holds that *N* ^*u*^ = *μ*_0_ = *n*_leaf_, and a minimal set of unrestricted inputs is indicated in the left panel (green arrows). To the right is a sparse plot of *V*_0_. The columns of *V*_0_ form a basis for ${{\mathscr{N}}}_{\ell }(A)$. Each node with outdegree ≥2 is a separate dilation, and the null space is the union of *d* = 5 structurally disjoint subspaces that are associated to the root and the dilations. A minimal set of unilateral controls is indicated in the left panel (red and blue arrows). For a RDT, *N* ^*r*^ does not depend on the numerical values of the edge weights, but the signs of the controls do. (**b**) Edges are added between nodes on the same layer. The tree structure is lost and the network is a DAG. By adding edges this way, longer paths are created. The depth *h*_max_ is increased and *μ*_0_ is reduced. Both *N* ^*r*^ and *N* ^*u*^ are reduced. (**c**) Descending edges are added to the original RDT. Neither *h*_max_ nor *μ*_0_ is altered and *N* ^*u*^ is unchanged. However, *d* is reduced and the lower bound *N* ^*r*^ ≥ *μ*_0_ + *d* is achieved.

Figure 3(a) shows *N* ^*r*^ and *N* ^*u*^ for a RDT. In Fig. 3(b), the RDT is extended with additional “non-ascending” edges between the nodes. This transforms the tree into a DAG. The density and the depth *h*_max_ have increased, making controllability easier to achieve. Both *N* ^*r*^ and *N* ^*u*^ are reduced while *η* is essentially unchanged. In Fig. 3(c), the RDT of Fig. 3(a) is instead extended with strictly “descending” edges added from the hubs. Interestingly, this makes unilateral controllability easier but not unrestricted controllability, and *η* is reduced. This can be understood from topological considerations and the structure of ${{\mathscr{N}}}_{\ell }(A)$ as explained in the next paragraph.

### Topology induced bound on the minimum number of unilateral controls

Since the columns of ${V}_{0}^{T}B$ have *μ*_0_ rows, the positive span condition means that ${V}_{0}^{T}B$ must have no less than *μ*_0_ + 1 columns. Since each column corresponds to one unilateral control input, *μ*_0_ + 1 is a lower bound on *N* ^*r*^. This lower bound can however be refined from the analysis of the network topology.

Let *d* be the number of roots and dilations in the graph ${\mathscr{G}}(A)$. As already mentioned, any root/dilation generates one or several zero eigenvalues of *A* with corresponding left eigenspaces. Denote by *σ*_*i*_, *i* = 1, …, *d*, the dimension of the eigenspace generated by the *i*:th root/dilation. For instance a root generates an eigenspace of dimension *σ*_*i*_ = 1, while dilations can be of different sizes and generate eigenspaces of arbitrary dimensions. The left null space of any matrix *A* representing a network is the union of these eigenspaces, hence ∑_*i*_ *σ*_*i*_ = *μ*_0_ (see the SI).

When *V*_0_ is constructed from a sparse basis of ${{\mathscr{N}}}_{\ell }(A)$, then it has a particular structure^23–25^. Columns that originate from different roots/dilations are structurally disjoint, i.e. the positions of their non-zero entries are non-overlapping. See Fig. 3 for an illustration of *V*_0_ for the studied RDT/DAGs. The structure of *V*_0_ propagates to the matrix ${V}_{0}^{T}B$ to which the positive span condition applies. Consequently, the unilateral control input selection problem becomes separable with respect to the different roots and dilations. In order to meet the positive span condition, the root/dilation *i* requires at least *σ*_*i*_ + 1 unilateral controls. When summing over all of them this leads to the refined lower bound *N* ^*r*^ ≥ *μ*_0_ + *d*.

When descending edges are added to the RDT as in Fig. 3(c), the number of subspaces, *d*, is reduced although *μ*_0_ unchanged. Hence the lower bound on *N* ^*r*^ is reduced but not the lower bound on *N* ^*u*^. See the SI for a full derivation of the lower bound.

The properties of these simple examples can help our understanding of more complex large scale networks.

## Results

In this section we study the minimal sets of unilateral resp. unrestricted control inputs that are required for controllability of random Erdős–Rényi networks^26^ and directed scale-free networks^27, 28^ with random edge weights. Also a number of real-world networks are studied. Controllability with unrestricted control inputs can also be addressed by the structural controllability framework^7^. When the edge weights are sampled from a continuous distribution as for instance a normal distribution, all the non-zero eigenvalues of *A* are generically simple^29^. On the contrary, the multiplicity of the zero eigenvalue is determined by the topology of the network, and it is generically invariant to the values of the numerical entries of *A*, see SI for more information. This implies that although controllability with unilateral controls is not a generic property in the sense of structural controllability, the problem is to a high degree “structural” (and generic). More precisely:

(i) Since all real non-zero eigenvalues are simple, for them the positive span condition of Theorem 1 leads to a set-cover problem.
(ii) Structural properties of ${{\mathscr{N}}}_{\ell }(A)$ limit the number of unilateral controls required to solve the problem of positively spanning sets that appear when *μ*_0_ > 1.

The first problem may be solved for all non-zero real eigenvalues simultaneously with only a few (but not less than two) unilateral control inputs. The second problem is more complicated but we will see in the following sections that the lower bound *μ*_0_ + *d* is often achieved.

A special case is when there is no zero eigenvalue (or it is simple) and the network has a node from which there exists a path to all other nodes. Then the network can be controlled with a single unrestricted control input. For instance a full network with random edge weights meets these conditions. On the other hand, the number of unilateral control inputs required for controllability of such a network can be either one or two. If *A* has one or more real eigenvalues (which is the general case for random networks, more details below) then two unilateral control inputs are required. However, if all eigenvalues are complex then one is sufficient. An example of a class of networks for which all eigenvalues of *A* are either zero or purely imaginary is given by skew-symmetric adjacency matrices, i.e. *a*_*ij*_ = −*a*_*ji*_. Hence, a full skew-symmetric network is controllable with only one unilateral control input.

When ${\mathscr{G}}(A)$ contains a dilation then the geometric multiplicity of the zero eigenvalue (and hence ${\rm{\dim }}{{\mathscr{N}}}_{\ell }(A)$) becomes larger than 1 and the unilateral controllability problem more interesting to investigate.

### Erdős–Rényi networks

Let *P*_in_(*k*_in_) be the indegree distribution and *P*_out_(*k*_out_) the outdegree distribution of a network. A random Erdős–Rényi network with *n* nodes and edge-probability *p* has *P*_in_(*k*_in_) = *P*_out_(*k*_out_) ~ Pois(*np*) where Pois(*np*) is the Poisson distribution with expected value *np*.

**Figure 4:**
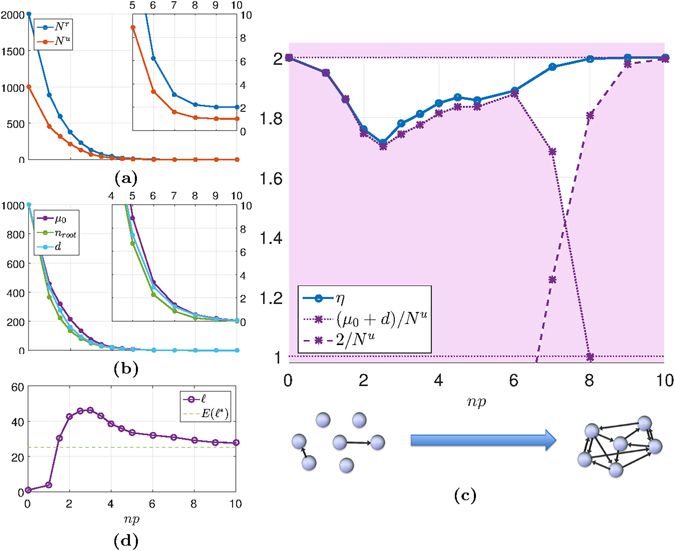
Unilateral controllability of Erdős–Rényi networks. Networks of size *n* = 1000 are generated with different edge-probabilities, *p*. For each configuration, 1000 random networks are generated with edge weights sampled from a normal distribution. Averaged values are shown. Note that the x-axis shows *np*, which is the expected indegree/outdegree for the nodes. (**a**) Both *N* ^*u*^ and *N* ^*r*^ decrease as *p* increases. *N* ^*u*^ follows *μ*_0_ (shown in (**b**)). (**b**) The dimension of ${{\mathscr{N}}}_{\ell }(A)$, *μ*_0_, the number roots *n*_root_ and the number of structurally disjoint subspaces *d* are shown. The networks in the left-end side of the plot have few edges and many roots. As the networks become more dense, the number of roots decreases and instead dilations appear in the networks. (**c**) The ratio *η* with its lower and upper bounds are shown. For *np* ≤ 6, *η* is always close to its lower bound (*μ*_0_ + *d*)/*N* ^*u*^ i.e. the extra *N* ^*r*^ − *N* ^*u*^ controls needed for unilateral controllability are due to the roots and dilations. When the networks become sufficiently dense there are neither roots nor dilations. Then *N* ^*r*^ approaches 2, a lower bound that comes from the presence of simple real eigenvalues in the adjacency matrix *A*. (**d**) The number of real eigenvalues $\ell$ approaches the constant level predicted by the theory as *p* increases.

Figure 4 shows results on controllability with unilateral and unrestricted control inputs for random Erdős–Rényi networks with *n* = 1000. The number *N* ^*r*^ is compared to *N* ^*u*^ in Fig. 4(a). The ratio *η* = *N* ^*r*^/*N* ^*u*^ is shown in Fig. 4(c). This plot also shows the different bounds that apply to *N* ^*r*^ and thus also to *η*: The lower bounds are *N* ^*r*^ ≥ *N* ^*u*^, *N* ^*r*^ ≥ *μ*_0_ + *d* and *N* ^*r*^ ≥ 2. The latter holds when *A* has least one real eigenvalue. The only upper bound is *N* ^*r*^ ≤ 2*N* ^*u*^ i.e. *η* ≤ 2. The null space is characterized in Fig. 4(b) by its dimension, *μ*_0_, the number of roots, *n*_root_, and *d* (the sum of *n*_root_ and the number of dilations).

The dimension of the null space decreases with increasing edge probability, and both *N* ^*u*^ and *N* ^*r*^ follow (Fig. 4(a)). For structural controllability the minimal number of unrestricted control inputs is mainly determined by the node degree distribution and dense networks requires less control inputs^4^. Our results suggest that this is also the case for unilateral controls.

The ratio *η* is close to 2 for very sparse networks. This is explained by the high number of roots in such networks. Almost the entire null space is generated by root nodes and the interval *μ*_0_ + *d* ≤ *N* ^*r*^ ≤ 2*N* ^*u*^ is tight. The ratio *η* follows its lower bound (*μ*_0_ + *d*)/*N* ^*u*^ closely as long as there exist roots or dilations in the networks (and *μ*_0_ ≥ 1), see Fig. 4(c). Hence, the most important factor for controllability with unilateral inputs is rather the network topology than the numerical values of the edge weights in this region. Moreover, the lower bound is in many cases achievable.

As can be seen in Fig. 4(c), for a significant range (roughly ^1, 6^), *η* behaves nontrivially: it drops from its upper bound 2 and its minimum is *η* ≈ 1.7. The drop occurs when *d* is significantly below *μ*_0_, meaning that the null space includes a number of subspaces with dimension ≥2. This is explained by the appearance of dilations; hubs with high outdegree or DAG-like submotifs. These structures often require only a few more unilateral inputs than unrestricted inputs in order to achieve controllability (as in Fig. 3).

In Fig. 4(d), we see that the number of real eigenvalues, $\ell$, approaches a constant level when *p* is increased. This is consistent with the asymptotic expression for the number of real eigenvalues of random matrices^30^: If $E({\ell }^{\ast })$ is the expected number of real eigenvalues of a full *n*-by-*n* random matrix whose elements are independent variables of a normal distribution, then $E({\ell }^{\ast })/\sqrt{n}=\sqrt{\mathrm{2/}\pi }$ as *n* → ∞. For the value of *n* = 1000 used in our numerical examples, even though *A* is sparse, we can see in Fig. 4(d) that the number of real eigenvalues of *A* approaches $E({\ell }^{\ast })$ as the density is increased. (Notice that the most dense networks in our simulations are still very sparse.)

Since each real eigenvalue entails a condition for unilateral controllability according to Theorem 1, one could think that the number of real eigenvalues influences the minimal number of unilateral controls. But since all non-zero real eigenvalues are simple, the positive span condition is met for several or even all simple real eigenvalues with very few unilateral controls. Even two controls are enough when the network consists of one strongly connected component, as in the example of Fig. 2(a). Hence when *A* has at least one real eigenvalue we have the third lower bound on *N* ^*r*^: *N* ^*r*^ ≥ 2 (or *η* ≥ 2/*N* ^*u*^). This bound is very conservative in most cases, but as the network density increases and the null space disappears (at *np* ~ 8) this becomes in fact the limiting bound on *N* ^*r*^, see Fig. 4(c).

Corresponding results are presented for smaller networks (*n* = 100) in Fig. S2. Since the results are essentially the same, we can conclude that our observations are valid regardless of the size of the Erdős–Rényi networks.

### Directed scale-free networks

**Figure 5:**
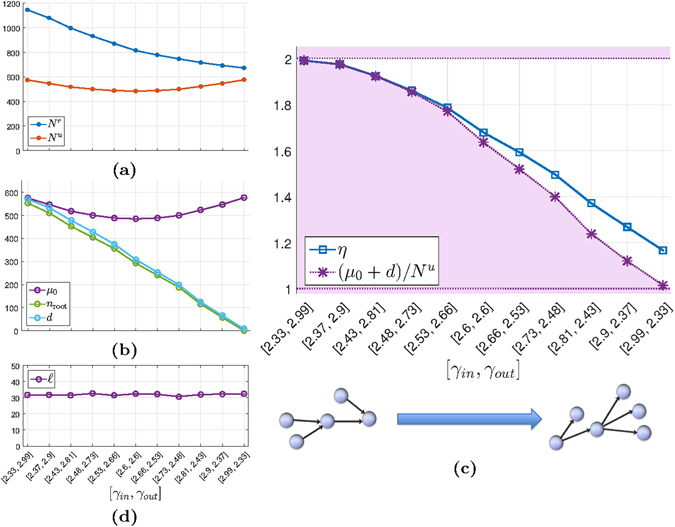
Unilateral controllability of scale-free networks. Networks of size *n* = 1000 are generated with different indegree and outdegree exponents, *γ*_in_ and *γ*_out_. For each configuration of *γ*_in_ and *γ*_out_, 1000 random networks with edge weights sampled from a normal distribution are generated and the figures show their averaged values. The total number of edges is the same for all configurations. (**a**) *N* ^*u*^ follows *μ*_0_ (shown in (**b**)) and is relatively constant for all configurations while *N* ^*r*^ drops significantly when the networks are shifted from indegree-dominated to outdegree-dominated. (**b**) *μ*_0_, *n*_root_ and *d* are shown. In the left-end side of the plot ${{\mathscr{N}}}_{\ell }(A)$ is essentially generated by the many roots present in these networks. As the degree exponents are shifted (from left to right of the plot), the roots tend to disappear and instead dilations add to the dimension of ${{\mathscr{N}}}_{\ell }(A)$. At the right end of the plots ${{\mathscr{N}}}_{\ell }(A)$ is essentially generated by one or a few large dilations, hence *d* ≪ *μ*_0_. (**c**) *η* with its upper and lower bounds are shown. Moving from left to right the lower bound (*μ*_0_ + *d*)/*N* ^*u*^ decreases and *η* follows it. The bound is however not achieved, which in part can depend on the heuristics that is used to compute *N* ^*r*^. (**d**) The number of real eigenvalues $\ell$ is fairly constant across degree distributions (the total density of edges is fixed).

The indegree and outdegree distributions of a directed scale-free network follow power-laws, ${P}_{{\rm{in}}}({k}_{{\rm{in}}})\propto {k}_{{\rm{in}}}^{-{\gamma }_{{\rm{in}}}}$ and ${P}_{{\rm{out}}}({k}_{{\rm{out}}})\propto {k}_{{\rm{out}}}^{-{\gamma }_{{\rm{out}}}}$ respectively. For real-world networks the degree exponents are typically in the range *γ*_in_, *γ*_out_ ∈ [2, 3]. The directed scale-free network model suggested in Bollabás *et al*.^31^ is used to generate random networks. It implements a preferential attachment process in which nodes and edges are added iteratively depending on the indegree and outdegree of existing nodes. By adjusting the model parameters that govern the indegree and outdegree distributions we can effectively tune to what extent the edges of the hubs of the network are directed outwards or inwards. When comparing *N* ^*r*^ with *N* ^*u*^ for directed scale-free networks, both the number of nodes and the total number of edges are kept constant and only the indegree and outdegree distributions are changed. Figure 5 illustrates the results on networks with *n* = 1000.

While *N* ^*u*^ varies relatively little, *N* ^*r*^ decreases significantly when the degree exponents are shifted so that the hubs have larger outdegree than indegree (the right-end of the plots). This result is coherent with previous examples and Fig. 2(c): a hub with only outgoing edges is an extreme case of *γ*_out_ ≪ *γ*_in_.

The characteristics of the null space in Fig. 5(b) can explain the changes in *N* ^*r*^: Networks with indegree-dominated hubs (left end of the plots) have a large number of roots, hence *d* and the bound *N* ^*r*^ ≥ *μ*_0_ + *d* are high. As the degree exponents shift, the roots disappear but instead dilations add to the dimension of the null space which remains high. This shift matters little when unrestricted control inputs are used as the lower bound *N* ^*u*^ ≥ *μ*_0_ is for most cases achieved either way. However, it matters when unilateral control inputs are used. The rightmost configuration (with *γ*_in_ = 2.99 and *γ*_out_ = 2.33) has, besides a few roots, normally only one large dilation that accounts for almost the whole null space. The effect is that *d* drops and so does *η*, meaning that controllability can be achieved with only a few more unilateral controls than unrestricted controls. The lower bound *η* ≥ (*μ*_0_ + *d*)/*N* ^*u*^ is however not achieved in our simulations. This can at least partially depend on the heuristics used to compute *N* ^*r*^. The number of real eigenvalues is about the same for all configurations, see Fig. 5(d).

The observations we have done here apply to directed scale-free networks of different sizes. Corresponding results are presented for smaller networks (*n* = 100) in Fig. S3.

### Analysis of real-world networks

**Figure 6:**
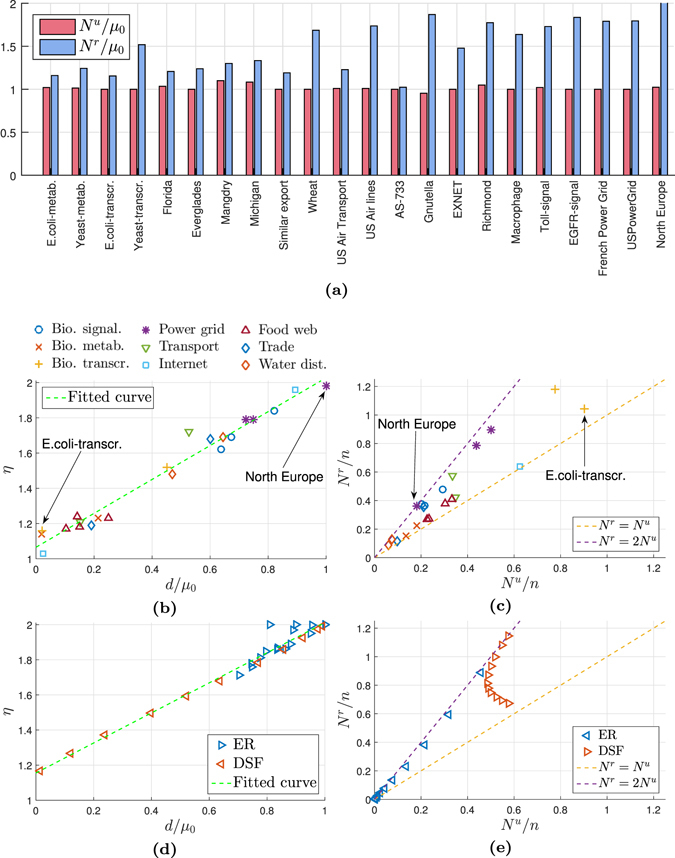
(**a**) The lower bound *N* ^*u*^ ≥ *μ*_0_, or *N* ^*u*^/*μ*_0_ ≥ 1 is in most cases achieved for the real-world networks in Table 3. (**b**) Here *η* is plotted against the ratio *d*/*μ*_0_ ∈ [0, 1] for the networks of Table 3. This ratio reflects in what extent ${{\mathscr{N}}}_{\ell }(A)$ is generated by roots and dilations. A linear regression curve is fitted to the data-points. The results for the different networks within a specific category are in most cases similar. (**c**) In this plot *N* ^*r*^ and *N* ^*u*^ are normalized with the network size. The admissible region *N* ^*u*^ ≤ *N* ^*r*^ ≤ 2*N* ^*u*^ is the area between the dashed lines. Here we can see that some networks require a large number of unrestricted or unilateral control inputs (far from the origin) although the ratio *η* is close to one (near the orange line). (**d**) The same plot as in (**b**) but for the Erdős–Rényi and directed scale free networks of Figs 4 and 5. There is one marker for each configuration. The outliers in the top right corner are dense Erdős–Rényi networks for which the null space parameters are of little importance in computing *η*. (**e**) Same as (**c**) but for the Erdős–Rényi and directed scale free networks.

A number of real networks taken from broadly different contexts (see the SI for details and references) have been analyzed with respect to the number of unilateral or unrestricted control inputs that are required to achieve controllability. When no information is available about the edge weights, these have been sampled from a normal distribution. The results are summarized in Table 3 and Fig. 6.

**Table 3:** Network characteristics and controllability results for the real-world networks considered in this paper.

Network type	Name	Nodes	Edges	*N* ^*r*^	*N* ^*u*^	$\frac{{{\boldsymbol{\mu }}}_{{\bf{0}}}{\boldsymbol{+}}{\boldsymbol{d}}}{{{\boldsymbol{N}}}^{{\boldsymbol{u}}}}$	*η*
Biology, transcr.	*E. coli*-transcr.	1623	3620	1692	1466	1.13	1.16
	Yeast-transcr.	664	1064	759	500	1.45	1.52
Biology, signal.	EGFR-signal	329	852	123	67	1.84	1.84
	Toll-signal	680	2204	249	147	1.65	1.69
	Macrophage	678	1582	300	185	1.60	1.62
Biology, metab.	Yeast-metab.	780	4420	174	142	1.21	1.23
	*E. coli*-metab.	757	6116	116	102	1.02	1.14
Power grid	North Europe	236	320	85	43	1.95	1.98
	USPowerGrid	4941	6591	3887	2166	1.72	1.79
	French Power Grid	1888	2531	1692	945	1.75	1.79
Transport	US Air lines	332	2126	191	111	1.51	1.72
	US Air traffic	1206	13106	511	420	1.15	1.21
Internet	Gnutella	6301	20777	8047	4106	1.94	1.96
	AS-733	3015	10312	1928	1883	1.03	1.03
Food-web	Florida	128	2106	35	30	1.10	1.17
	Michigan	39	221	16	13	1.23	1.23
	Mangdry	97	1491	26	22	1.14	1.18
	Everglades	69	916	26	21	1.14	1.24
Trade	Similar export	866	2532	100	84	1.19	1.19
	Wheat	166	1789	59	35	1.60	1.69
Water dist.	EXNET	1893	4832	167	113	1.47	1.48
	Richmond	865	1870	110	65	1.57	1.69

See SI for data sources. Just as for Erdős–Rényi networks and directed scale-free networks, *η* is in many cases close to the lower bound (*d* + *μ*_0_)/*N* ^*u*^.

As shown in Table 3, the values of *η* for these networks cover the entire range [1, 2]. This variation in *η* and the correlation between *η* and the number of roots and dilations in the networks is also shown in Fig. 6(b). The correlation between *η* and *d*/*μ*_0_ is 0.96 for the considered networks, hinting that the ratio *d*/*μ*_0_ is a good explanatory variable for *η*. This can be understood by observing that both *N* ^*r*^ and *N* ^*u*^ tend to meet their lower bounds: from ${N}^{u}\simeq {\mu }_{0}$ (see Fig. 6(a)) and ${N}^{r}\simeq {\mu }_{0}+d$ it follows $\eta \simeq ({\mu }_{0}+d)/{\mu }_{0}=1+d/{\mu }_{0}$.

At one end of the spectrum (*η* near 1) we find networks from certain categories like metabolic networks and food webs. Also the *E. coli-transcr*. gene regulatory network has a very low ratio *η*. This last network is very sparse and with low edge density. Figure S4(a) shows the graph of the network, and Fig. S4(b) its indegree and outdegree distributions. It requires a large number of control inputs: almost every node must be separately controlled when unrestricted inputs are used, see Fig. 6(c). However, it turns out that the use of unilateral controls does not make controllability significantly more difficult to achieve. The graph of the network consists of one (weakly) connected component and has in essence a DAG-like structure. It has a few roots and hubs that each have hundreds of outgoing edges, while most of the other nodes are leaves with no outgoing edge at all. Its maximal DAG subgraph includes all its nodes and 97% of the edges. The depth *h*_max_ is low, hence it is a rather “flat” network, with only a few layers in the core DAG structure and short paths between the nodes. The relative “flatness” of the network can be related to the rooted directed trees studied earlier, see Table 2 and Fig. 3. There it was concluded that a flat RDT results in lower values of *η*, in contrast to networks with many layers. Furthermore, the value of *η* is close to the topologically induced lower bound (*μ*_0_ + *d*)/*N* ^*u*^, see Table 3.

At the other end of the spectrum (i.e. *η* near 2) we have other categories of networks, such as the power grids. Consider for instance the *North Europe* power grid. The graph of this network is shown with its unilateral controls in Fig. S5. Also this network is very sparse, but in contrast to the *E. coli-transcr*. network, it has several long paths connecting distant nodes. It was observed in our earlier analysis of DAGs that when there are long paths in a network, then relatively few unrestricted control inputs are required for controllability. This observation holds also for the North Europe grid network, see Fig. 6(c). Furthermore, the network has no significant hubs but quite many roots. These roots typically correspond to power plants that are located in remote areas.

## Discussion

In an effort to render more realistic the problem of controlling complex networks, in this paper we have taken inspiration from classical control literature in order to formulate a control problem in which the inputs are constrained in sign, i.e. they can only “push” or “pull”. This is motivated by the fact that unilateral controls are more common than bidirectional controls in many contexts, such as for instance biological networks (drugs act as inhibitors or activators, not both), food webs (a species can be added by stocking/feeding or selectively reduced by hunting/fishing), traffic (cross-lights and speed limits can reduce traffic), power grids (generators can inject power, loads can consume power), trade (subsidies of domestic production, import restrictions and tolls), see Table 1 for more details. The use of classical conditions, such as Brammer’s controllability condition, passes through their reformulation in a more computationally-oriented form. The development of an algorithm that identifies a near-minimal set of unilateral control inputs for a given network has enabled us to study in a systematic way various categories of random networks, as well as real-world networks.

The conditions for unilateral controllability are formulated algebraically in terms of eigenspaces of the system matrix *A* and positively spanning sets. With random edge-weight assignments, the unilateral controllability problem is to a high degree structural, since non-zero eigenvalues are simple and the null space is determined by the topology of the network. What is *not* structural is whether the non-zero eigenvalues are real or complex conjugate. This property is irrelevant for structural controllability, but it matters for unilateral controllability. For random matrices and *n* → ∞, the number of real eigenvalues can be estimated^30^. For other network topologies an analytic estimation of the number of real non-zero eigenvalues is missing. Our numerical calculations (Figs 4(d) and 5(d)) suggest that even for size *n* ~ 10^3^ such number is stable across numerical realizations. Also the *N* ^*r*^ that follows is very stable for all topologies (in Figs 4 and 5 variations are never shown because they are always very small). Throughout this study we notice that the topology of a network is much more crucial than the numerical values of the edges, in order to understand what unilateral control inputs should be selected. The topology enters in the unilateral controllability conditions through the left null space ${{\mathscr{N}}}_{\ell }(A)$. What determines *N* ^*r*^ is not only the dimension of the null space *μ*_0_ but also in what extent ${{\mathscr{N}}}_{\ell }(A)$ is generated by roots or dilations, i.e. the parameter *d*. The results we achieved with our algorithm often meet the lower bound *N* ^*r*^ ≥ *μ*_0_ + *d*, and the relative cost of unilateral controllability, *η*, is well explained by the ratio *d*/*μ*_0_, see Fig. 6(b,d).

As a by-product, we obtain a valid topological interpretation also for (unrestricted) controllability. In structural controllability it is known that roots and dilations of the network can be directly mapped to the choice of certain nodes as driver nodes. Algebraically these properties relate to the dimension of the null space, *μ*_0_, and *μ*_0_ almost completely determines *N* ^*u*^ (see Fig. 6(a) for the real-world networks and Fig. S6 for Erdős–Rényi and directed scale free networks). Since, as already mentioned, the dimension of the null space does not depend on the numerical entries of *A*, the same *N* ^*u*^ obtained through structural controllability is obtained generically through random edge weights assignments^7^. Notice that the specific continuous probability distribution from which the edge weights are drawn is irrelevant for our conclusion (i.e. non-zero eigenvalues remain simple also if we change distribution model^29^). This implies that if for instance we replace the normal distributions used here with uniform distributions, we can obtain networks with nonnegative edge weights, a more realistic network model for some applicative contexts. Our results apply unchanged also in this case.

An intrinsic limitation of the conditions developed in this paper (as well as of other approaches like structural controllability) is that the dynamics of the network is assumed to be linear. Even when the linear dynamics is the result of a linearization around an equilibrium point *x*_*o*_, in order to preserve the unidirectionality of our controls we have to assume that the nominal value of the input at the equilibrium point is *u*_*o*_ = 0. In other words, extended linearizations around a pair (*x*_*o*_, *u*_*o*_) in which *u*_*o*_ ≠ 0 lead to loss of unilaterality of the inputs when computed around *u*_*o*_. It is worth pointing out, however, that for complex networks considering an equilibrium point which is a function of a continuously applied input is an unlikely situation for most of the classes of networks considered in this study (a biological network can function also without a drug, traffic can flow also without cross lights or tolls, etc.).

The binary (yes/no) question of controllability has several shortcomings. On the one hand it can be argued that controllability is an unnecessarily ambitious goal^32^. In many applications it is enough to control a subset of the nodes, i.e. achieve controllability in a subspace of ${{\mathbb{R}}}^{n}$. On the other hand, a network that is controllable according to the mathematical definition is not necessarily controllable in practice. It could for instance be the case that almost infinite levels of energy are required to reach certain states^33–36^. Different metrics for the control effort are suggested in the literature. When control inputs are unrestricted, these are often based on the controllability gramian. However, with constrained inputs the problem becomes significantly more difficult as the least energy path may violate unilaterality. Even for small-scale control systems systematic solutions are almost impossible to compute^37, 38^.

## Electronic supplementary material

Supplementary information for Controllability of complex networks with unilateral inputs
