# Supplementary material for: Controllability of complex networks with unilateral inputs
Source: Sci Rep. 2017 May 12;7:1824. doi: 10.1038/s41598-017-01846-6 (PMC5432006; doi:10.1038/s41598-017-01846-6)
Supplement: Supplementary file 1 — Supplementary information for Controllability of complex networks with unilateral inputs [file 41598_2017_1846_MOESM1_ESM.pdf]

# Supplementary information for Controllability of complex networks with unilateral inputs

Gustav Lindmark and Claudio Altafini*

Division of Automatic Control, Dept. of Electrical Engineering, Linköping University, SE-58183, Linköping, Sweden.

## Contents

1 **Description of the real-world networks used in this study** **2**

2 **Positive linear dependence** **3**

3 **Conditions for unilateral controllability** **4**

4 **Construction procedure for unilateral control inputs** **7**

5 **Topology induced bound on the minimum number of unilateral controls** **10**

6 **Unilateral controls for some specific network structures** **13**

## List of Figures

S1 . . . . . . . . . . . . . . . . . . . . . . . . . . . . . . . . . . . . . . . 19
S2 . . . . . . . . . . . . . . . . . . . . . . . . . . . . . . . . . . . . . . . 20
S3 . . . . . . . . . . . . . . . . . . . . . . . . . . . . . . . . . . . . . . . 21
S4 . . . . . . . . . . . . . . . . . . . . . . . . . . . . . . . . . . . . . . . 22
S5 . . . . . . . . . . . . . . . . . . . . . . . . . . . . . . . . . . . . . . . 23
S6 . . . . . . . . . . . . . . . . . . . . . . . . . . . . . . . . . . . . . . . 24
S7 . . . . . . . . . . . . . . . . . . . . . . . . . . . . . . . . . . . . . . . 25

*Corresponding author: C. Altafini. E-mail: claudio.altafini@liu.se

# 1 Description of the real-world networks used in this study

Information about the real-world networks considered in this study is compiled here.

- Biology, transcriptional networks
  - *E.coli-transcr.:* Gene regulatory network of the *E.coli*, downloaded from *RegulonDB* database (http://regulondb.ccg.unam.mx). Only the largest connected component is used.
  - *Yeast-transcr.:* Gene regulatory network of *S.cerevisiae* originally developed in Milo et al.[1] Only the largest connected component is used.
- Biology, signaling networks
  - *EGFR-signal:* The network for the Epidermal Growth Factor receptor pathway. It was created by Oda et al.[2]
  - *Toll-signal:* Signaling network for the *Toll-like*-receptor. Assembled from Oda et al.[3]
  - *Macrophage:* The molecular interaction map of a macrophage obtained by Oda et al.[4]
- Biology, metabolic networks
  - *Yeast-metab:* Metabolic network of the yeast *S.cerevisiae*. Assembled from the list of reactions in Förster et al.[5]
  - *E.coli-metab:* Metabolic network of E.coli, from Reed et al.[6]
- Power Grid
  - *USPowerGrid:* This network represents the Power Grid of the Western States in the United States. It is described in Watts et al.[7] and is available at http://cdg.columbia.edu/cdg/datasets.
  - *North Europe:* Power grid retrieved from Menck et al.[8]
  - *French Power Grid:* This network is available in the MATPOWER Matlab toolbox for power flow problems, http://www.pserc.cornell.edu/matpower. It is described in Josz et al.[9]
- Internet
  - *Gnutella:* Peer-to-peer file sharing network from Ripeanu et al.[10]
  - *AS-733:* A snapshot of the structure of the Internet at the level of Autonomous Systems. The network is described in Karrer et al.[11]

These networks are part of the Stanford Large Network Dataset Collection[12].

- Food-web

  The food-web networks are described in Madenjian et al.[13] and Ulanowicz et al.[14] They are part of the Pajek collection[15].

- Transport

  - *US Air traffic:* Network that represents connected airports in the US. It is described on the website of Lev Muchnik, http://www.levmuchnik.net/Content/Networks/NetworkData.html, where it is also available for download.
  - *US Air lines:* This network is part of the Pajek collection[15].

- Trade

  - *Similar export:* Network of export products created by Hidalgo et al.[16]
  - *Wheat:* Network representing trade relationships between countries from the Food and Agricultural Organization of the United Nations, see Domenico et al.[17]

- Water distribution

  The water distribution networks *EXNET* and *Richmond* are described in A. Yazdani et al.[18]

# 2 Positive linear dependence

The theory of positive linear dependence provides a useful framework for our controllability analysis with unilateral inputs. Some important results from Davis[19] are recalled here. A *positive combination* of vectors $a_1, \ldots, a_r$ is a linear combination $\theta_1 a_1 + \cdots + \theta_r a_r$ with $\theta_i \geq 0,\ i = 1, \ldots, r$. An algebraic equation $\theta_1 a_1 + \cdots + \theta_r a_r = 0$ with $\theta_i \geq 0$ is said a *positive relation*, and with $\theta_i > 0$ it is called a *strictly positive relation.*

A *convex cone* $\mathcal{C}$ is the set of all positive combinations of a finite set of vectors, not all zero

$$\mathcal{C} = \left\{ \sum_{i=1}^{r} \theta_i a_i \mid \theta_i \in \mathbb{R}^+ \right\}. \tag{1}$$

The set of vectors $a_1, \ldots, a_r$ is said to *positively span* the convex cone $\mathcal{C}$.

Given a matrix $M$, let $M[k]$ denote the $k$-th column of $M$ and $M[i,j]$ denote the element on row $i$ and column $j$ of the matrix $M$. When used on a vector, $a[k]$ denotes the $k$-th element of $a$. For $M \in \mathbb{R}^{p \times q}$, $\mathcal{C}(M) \subseteq \mathbb{R}^p$ is the convex cone of positive combinations of its columns.

The vectors $a_1, \ldots, a_r \in \mathbb{R}^n$ positively span $\mathbb{R}^n$ if for any $x \in \mathbb{R}^n$, $\exists\ \theta_i \geq 0$ s.t. $x = \theta_1 a_1 + \cdots + \theta_r a_r$. To avoid confusion, *linearly span* is used for spanning in the usual sense. The following theorems from Davis[19] will be useful:

**Theorem 1.** *Let $\{a_1, \ldots, a_r\}$ linearly span $\mathbb{R}^n$. Then the following are equivalent:*

*i) $\{a_1, \ldots, a_r\}$ positively span $\mathbb{R}^n$.*

*ii) There is some strictly positive relation between the $a_i$, $i = 1, \ldots, r$.*

To positively span $\mathbb{R}^n$, at least $n+1$ vectors are needed. Given the two vectors $a_1$ and $a_2$, denote $\langle a_1, a_2 \rangle$ their Euclidean inner product.

**Theorem 2.** *The vectors $a_1, \ldots, a_r$ positively span $\mathbb{R}^n$ if and only if, for every non-zero $b \in \mathbb{R}^n$, there is an $i \in \{1, \ldots, r\}$ such that $\langle b, a_i \rangle > 0$.*

Theorem 2 implies that if $a_1, \ldots, a_r$ *do not* positively span $\mathbb{R}^n$, then there is a $b$ such that $\langle b, a_i \rangle \leq 0\ \forall i \in \{1, \ldots, r\}$. Given a set of vectors $a_1, \ldots, a_r \in \mathbb{R}^n$, the problem of determining if they positively span $\mathbb{R}^n$ can be done by solving a linear optimization problem[20].

# 3 Conditions for unilateral controllability

Consider the system

$$\dot{x} = Ax + Bu, \tag{2a}$$
$$u \in \Omega, \tag{2b}$$

with $A \in \mathbb{R}^{n \times n}$ and $B \in \mathbb{R}^{n \times m}$. The *admissible controls* are all vector functions $u(t)$ taking value in the *control restraint set*, $\Omega \subset \mathbb{R}^m$. The system (2) is *controllable* if for each pair of $x_0$ and $x_1$ in $\mathbb{R}^n$, there exist an admissible control, $u(t) \in \Omega$, defined on some finite interval $0 \leq t \leq t_1$, which steers $x_0$ to $x_1$. The *reachable set* $\mathcal{R}(t)$ is the set of all points in $\mathbb{R}^n$ to which the origin can be steered at time $t$ by an admissible control $u \in \Omega$. The reachable set $\mathcal{R}_\infty$ is the union of $\mathcal{R}(t)$ over all positive $t$. The system is *null-controllable* if there exists an open set $\Gamma$ in $\mathbb{R}^n$ containing the origin for which any $x_0 \in \Gamma$ can be controlled to $x_1 = 0$ in finite time.

Conditions for controllability of systems with constrained inputs are derived in Brammer[21] and further investigated in Heymann et al.[22] The main theorem states that the following conditions are necessary and sufficient for null-controllability of (2):

**Theorem 3.** *(Brammer[21]) Consider the system* (2) *satisfying the following conditions:*

*i) There exists $u \in \Omega$ satisfying $Bu = 0$.*

*ii) The convex hull of* $\Omega$ *has nonempty interior in* $\mathbb{R}^m$*.*

*Then* (2) *is null-controllable if and only if*

*iii) The matrix* $\begin{bmatrix} B & AB & A^2B & \dots & A^{n-1}B \end{bmatrix}$ *has rank* $n$*.*

*iv) There is no real left eigenvector* $v$ *of* $A$ *s.t.* $\langle v, Bu \rangle \leq 0 \ \forall u \in \Omega$*.*

A *real eigenvector* denotes an eigenvector associated with a real eigenvalue. An alternative but equivalent formulation of *iv)* in Theorem 3 is:

*iv') For any real left eigenvector* $v$ *of* $A$ *there must be a* $u \in \Omega$ *such that* $\langle v, Bu \rangle > 0$*.*

In this paper we study a system with unilateral controls and assume that each control input only acts on a single node either positively or negatively. Then the control restraint set is $\Omega = \mathbb{R}^m_+$, and $B$ has the structure

$$B = \begin{bmatrix} \pm e_{i_1} \dots \pm e_{i_m} \end{bmatrix} \tag{3}$$

with $e_i$ the $i$-th elementary vector, $i \in 1, \dots, n$.

For the system (2) with control restraint set $\Omega = \mathbb{R}^m_+$, condition *ii)* of Theorem 3 is met, and, since $u = 0$ is admissible, clearly also condition *i)* holds. Furthermore, null-controllability implies in this case controllability since the inputs can be arbitrarily scaled, enlarging the set $\Gamma$ to cover $\mathbb{R}^n$.

Condition *iv)* of Theorem 3 can be reformulated as follows. Let $\lambda_0 = 0$, $\lambda_1, \dots, \lambda_\ell$, be the distinct real eigenvalues of $A \in \mathbb{R}^{n \times n}$, and $\mu_i, \ i = 0, \dots, \ell$ their geometric multiplicity. Denote $\{v_{i,1}, \dots, v_{i,\mu_i}\}$ the linearly independent real eigenvectors that linearly span the eigenspace of $\lambda_i$. Assume that $B \in \mathbb{R}^{n \times m}$ is given. Define

$$V_i = [v_{i,1} \ v_{i,2} \dots v_{i,\mu_i}], \tag{4}$$

and

$$\rho_i = V_i^T B \in \mathbb{R}^{\mu_i \times m}, \quad i = 0, \dots, \ell. \tag{5}$$

From (4), any left eigenvector $v_i$ associated with $\lambda_i$, i.e. $v_i^T A = v_i^T \lambda_i$, can be written as a linear combination

$$v_i = V_i \alpha_i, \ \alpha_i \in \mathbb{R}^{\mu_i}. \tag{6}$$

**Lemma 1.** *For any left real eigenvector* $v$ *of* $A$ *there is a* $u \geq 0$ *such that* $\langle v, Bu \rangle > 0$*, if and only if the columns of* $\rho_i$ *positively span* $\mathbb{R}^{\mu_i}$ $\forall i = 0, \dots, \ell$*.*

*Proof.* "If part". Denote $\mathcal{V} = \{V_0\alpha_0, \dots, V_\ell \alpha_\ell\}$, $\alpha_i \in \mathbb{R}^{\mu_i}, \ i \in \{0, \dots, \ell\}$, the set of all left real eigenvectors of $A$. Selecting any eigenvector $v \in \mathcal{V}$, from (6),

$$\langle v, Bu \rangle = \langle \alpha_i, \rho_i u \rangle \tag{7a}$$

$$= \sum_{j=1}^{m} \langle \alpha_i, \rho_i[j] \rangle u[j]. \tag{7b}$$

By assumption any point in $\mathbb{R}^{\mu_i}$ is a positive combination of the columns of $\rho_i$ since they positively span $\mathbb{R}^{\mu_i}$. For example, given $\alpha_i$, there is a positive combination with $u \geq 0$ s.t. $\rho_i u = \alpha_i$. From (7a) we then have $\langle v, Bu \rangle = \langle \alpha_i, \alpha_i \rangle > 0$.

"Only if part". By contradiction, assume the columns of $\rho_i$ do not positively span $\mathbb{R}^{\mu_i}$. We will show that there is a $v \in \mathcal{V}$ for which $\langle v, Bu \rangle \leq 0,\ \forall u \geq 0$. Consider (7b) and use Theorem 2 with $\rho_i[j]$, $j = 1, \ldots, m$, instead of the vectors $a_1, \ldots, a_r$, and $\alpha_i$ instead of $b$. Since $\rho_i[j],\ j = 1, \ldots, m$, do not positively span $\mathbb{R}^{\mu_i}$, there is a $\alpha_i$ for which $\langle \alpha_i, \rho_i[j] \rangle \leq 0\ \forall j$. Used in (7b), this gives $\langle v, Bu \rangle \leq 0$ which is a contradiction. ■

Theorem 4 follows when Lemma 1 is used with Brammers conditions for controllability.

**Theorem 4.** *The system* (2) *is controllable with unilateral control inputs if and only if*

*i) The matrix* $\begin{bmatrix} B & AB & A^2B & \ldots & A^{n-1}B \end{bmatrix}$ *has rank* $n$.

*ii) The columns of* $V_i^T B$ *positively span* $\mathbb{R}^{\mu_i}$ $\forall i = 0, 1, \ldots, \ell$.

### Structure of the input matrix

When each control input acts on a single state, positively or negatively, the matrix $B$ has the form

$$B = \begin{bmatrix} B^+ & -B^- \end{bmatrix}, \text{ where} \tag{8a}$$
$$B^+ = \begin{bmatrix} e_{p_1} & e_{p_2} & \ldots & e_{p_{m^+}} \end{bmatrix}, \text{ and} \tag{8b}$$
$$B^- = \begin{bmatrix} e_{q_1} & e_{q_2} & \ldots & e_{q_{m^-}} \end{bmatrix}, \tag{8c}$$

where $e_i$ is the $i$-th elementary vector, $i \in 1, \ldots, n$. Define the index sets $\mathcal{P} = \{p_1, \ldots, p_{m^+}\}$ and $\mathcal{Q} = \{q_1, \ldots, q_{m^-}\}$, and consider the matrix $V$ as the combination of all real eigenvectors,

$$V = \begin{bmatrix} V_0 & \ldots & V_\ell \end{bmatrix}, \tag{9}$$

with $V_i,\ i = 0, \ldots, \ell$, as in (4). With the given form of $B$, we have

$$V^T B = \begin{bmatrix} \rho_0 \\ \vdots \\ \rho_\ell \end{bmatrix} = \begin{bmatrix} V^T[\mathcal{P}] & -V^T[\mathcal{Q}] \end{bmatrix}. \tag{10}$$

The notation $V^T[\mathcal{P}]$ denotes the matrix $\begin{bmatrix} V^T[p_1] & \ldots & V^T[p_m] \end{bmatrix}$ (and $V^T[\mathcal{Q}]$ analogously).

# 4 Construction procedure for unilateral control inputs

Assume that the system matrix $A$ is given. We want to construct a matrix $B$ such that the system $(A, B)$ is controllable with as few unilateral control inputs as possible. In the construction of $B$, we assume that we are free to add control inputs that act on any single state either positively or negatively.

### Greedy input selection with simple real eigenvectors

When all real eigenvalues are simple, then $V_i = v_{i_1},\ i = 0, \ldots, \ell$, and the rows of $V^T B$ are the row vectors $\rho_i \in \mathbb{R}^{1\times(m^+ + m^-)}$. When $\rho_i$ has only one row, its colums positively span $\mathbb{R}$ when there is both a positive and a negative entry. Notice that the input column $e_j,\ j \in \mathcal{P}$ (resp. $-e_r,\ r \in \mathcal{Q}$) corresponds to the column $V^T[j]$ (resp. $-V^T[r]$) in (10). From Theorem 4, a procedure to construct $B$ must make sure that all rows of $V^T B$ are *covered* with a positive and a negative entry. Furthermore, we seek a minimum number of control inputs that accomplishes that.

This problem can be formulated as a variant of the well known combinatorial *Set-Cover Problem*[23]: Given a set of elements $\mathcal{U}$ (called the universe) and a collection of sets, $\mathcal{S} = \{\mathcal{S}_1, \ldots, \mathcal{S}_r\}$ for which it holds that $\cup_{j=1}^r \mathcal{S}_j = \mathcal{U}$. The set-cover problem is to identify the smallest sub-selection of $\mathcal{S}$ whose union is $\mathcal{U}$. This is an NP-complete problem, meaning that finding the exact global minimum becomes unfeasible when the size of the system grows. Greedy heuristics can be used instead. Such methods do not guarantee optimality, but in general result in a good approximation.

Assume that an initial control input matrix $B$ and the corresponding sets $\mathcal{P}$ and $\mathcal{Q}$ are given (possibly empty). Then $B$ is extended by adding input columns one at a time, and $\mathcal{P}$ and $\mathcal{Q}$ are updated accordingly. At every step of the iteration, the control input with the highest *gain* $\gamma^* = \max\{\gamma_j^+, \gamma_r^-\}$ is selected, where the gain $\gamma_j^+$ (resp. $\gamma_r^-$) evaluates the contribution of the new positive control input candidate $b_j$ (resp. negative control input candidate $-b_r$). In formulas:

$$\begin{aligned}\gamma_j^+ &= \kappa\left(\begin{bmatrix} V^T[\mathcal{P} \cup j] & -V^T[\mathcal{Q}]\end{bmatrix}\right) \\ &\quad - \kappa\left(\begin{bmatrix} V^T[\mathcal{P}] & -V^T[\mathcal{Q}]\end{bmatrix}\right),\end{aligned} \tag{11}$$

where $\kappa(\cdot)$ is the sum of the number of rows with a positive entry and the number of rows with a negative entry. $\gamma_j^-$ is defined accordingly for extension with a negative control input. The procedure is summarized in Algorithm 1.

**Algorithm 1** Greedy Algorithm for Control Input Selection

0. Start with an initial $B$, and the corresponding sets $\mathcal{P}$ and $\mathcal{Q}$ (possibly empty).
1. For $j = \{1, \ldots, n\} \setminus \mathcal{P}$, calculate $\gamma_j^+$ and for $r = \{1, \ldots, n\} \setminus \mathcal{Q}$ calculate $\gamma_r^-$.
2. Select a control input with maximum gain, $\gamma^* = \max\{\gamma_j^+, \gamma_r^-\}$ i.e., $e_j$ for $j$ s.t. $\gamma_j^+ = \gamma^*$ or $-e_r$ for $r$ s.t. $\gamma_r^- = \gamma^*$. Update $B := \begin{bmatrix} B & e_j \end{bmatrix}$ and $\mathcal{P} := \{\mathcal{P} \cup j\}$, or $B := \begin{bmatrix} B & -e_r \end{bmatrix}$ and $\mathcal{Q} := \{\mathcal{Q} \cup r\}$.
3. Repeat from step 1 until condition *ii)* of Theorem 4 holds.

The procedure always terminates successfully in at most $2\ell$ iterations.
**Example:** Assume $A$ has three real left eigenvectors, grouped in the matrix

$$V^T = \begin{bmatrix} 1 & 0 & 0 \\ 1 & 1 & 0 \\ 0 & 1 & -1 \end{bmatrix}.$$

After three iterations of Algorithm 1, we have

$$B = \begin{bmatrix} e_1 & e_2 & -e_1 \end{bmatrix},$$
$$\mathcal{P} = \{1,\ 2\} \text{ and } \mathcal{Q} = \{1\}.$$

In the fourth iteration of Algorithm 1, at step 1, we should calculate $\gamma_3^+$, $\gamma_2^-$ and $\gamma_3^-$:

$$\gamma_3^+ = \kappa\left(\begin{bmatrix} 1 & 0 & 0 & -1 \\ 1 & 1 & 0 & -1 \\ 0 & 1 & -1 & 0 \end{bmatrix}\right) - \kappa\left(\begin{bmatrix} 1 & 0 & -1 \\ 1 & 1 & -1 \\ 0 & 1 & 0 \end{bmatrix}\right) = (3+3) - (3+2) = 1.$$

The extended matrix has three rows with positive entries and three rows with negative entries compared to the original matrix with three rows with positive entries but only two rows with negative entries. In the same manner, $\gamma_2^- = 1$ and $\gamma_3^- = 0$. Therefore we can select the input column $e_3$ and extend $B$ to $\begin{bmatrix} B & e_3 \end{bmatrix}$ and $\mathcal{P}$ to $\{\mathcal{P} \cup 3\}$ (step 2 in Algorithm 1). With this extension, we have

$$\begin{bmatrix} \rho_1 \\ \rho_2 \\ \rho_3 \end{bmatrix} = \begin{bmatrix} 1 & 0 & 0 & -1 \\ 1 & 1 & 0 & -1 \\ 0 & 1 & -1 & 0 \end{bmatrix}.$$

The condition *ii)* of Theorem 4 is met and Algorithm 1 has finished. Alternatively instead of $e_3$ one could have selected $-e_2$, obtaining the same result.

**Extension for multidimensional real eigenspaces**

From condition *ii)* of Theorem 4, positive controllability requires that the columns of $\rho_i$, $i = 0, \ldots, \ell$, positively span $\mathbb{R}^{\mu_i}$. This can be accomplished by the iterative construction of $B$ in Algorithm 1. However, the gain must be defined differently when $\rho_i$ is s.t. $\mu_i > 1$. From equation (9) and (10), extending $B$ to $\begin{bmatrix} B & e_j \end{bmatrix}$ implies $\rho_i := \begin{bmatrix} \rho_i & V_i^T[j] \end{bmatrix}$, $i = 0, \ldots, \ell$.

Consider a step in our iterative construction process. $B$ consists of the columns of the already selected inputs. $\mathcal{P}$ and $\mathcal{Q}$ are computed accordingly, and $\rho_i, i = 0, \ldots, \ell$, is calculated. Next, the contribution of extending $B$ to $\begin{bmatrix} B & e_j \end{bmatrix}$ must be evaluated. If the convex cone $\mathcal{C}(\rho_i) \subset \mathcal{C}\left(\begin{bmatrix} \rho_i & V_i^T[j] \end{bmatrix}\right)$ for any $i$, then including $e_j$ will bring us closer to meet condition *ii)* of Theorem 4.

In case there are several control inputs that enlarges $C(\rho_i)$ if included, a simple greedy selection strategy is used. Based on $m$ already selected control inputs, calculate the average direction of the vectors that positively span $\mathcal{C}(\rho_i)$,

$$\bar{\rho}_i = \frac{\sum_{r=1}^{m} \rho_i[r]/|\rho_i[r]|}{m}, \; i = 1, \ldots, \ell. \tag{12}$$

Then calculate the gain of extending $B$ with $e_j$ as

$$\hat{\gamma}_j^+ = \sum_{i=1}^{l} (1 - \langle \bar{\rho}_i, V_i^T[j] \rangle). \tag{13}$$

In (13), the inner product is a measure of distance between $V_i^T[j]$ and the barycentric direction of $\mathcal{C}(\rho_i)$. $B$ is extended with the control input that maximizes this distance, all cones considered. Control inputs that are acting negatively on a node are evaluated correspondingly. After each extension of $\rho_i$ one must evaluate whether $C(\rho_i)$ positively span $\mathbb{R}^{\mu_i}$. When the gain is calculated in this way, Algorithm 1 can be applied to multidimensional eigenspaces. Algorithm 1 will always terminate successfully.

Algorithm 1 provides a method to select inputs such that condition *ii)* of Theorem 4 is met. The rank condition, *i)* of Theorem 4, is the same as for the case of unrestricted control inputs. There are different methods to deal with this problem described in the literature. For instance in Commault et al.[24] this is done in a

structural sense, while in Yuan et al.[25] exact methods (PBH test) are used.

# 5 Topology induced bound on the minimum number of unilateral controls

The topology of a network is important to determine what control inputs that are needed for controllability. This is well investigated in the theory on structural controllability, where properties of the graph representing the network are directly mapped to the choice of certain nodes as driver nodes.

Although the conditions for unilateral controllability are formulated algebraically in terms of eigenspaces of the system matrix $A$ and positively spanning sets, the topology still enters in the equations through the left eigenspace of the zero eigenvalue $\lambda_0 = 0$, i.e. the left null space $\mathcal{N}_\ell(A)$. Roots and dilations in the directed graph $\mathcal{G}(A)$ are associated to the zero eigenvalues of $A$. By analyzing a topological property such as the structure of $\mathcal{N}_\ell(A)$ we are able to identify a lower bound on the number $N^r$ of unilateral control inputs that are required for controllability. It should be said that a zero eigenvalue may also appear "by coincidence" as a result of algebraic dependencies between the entries of $A$. When the entries of $A$ are drawn randomly, this however can happen only on a zero measure set, a case akin to the failure of a structural controllability method. This zero-measure set will not be considered in the remaining of this section.

#### Structure of the null space

Consider the graph $\mathcal{G}(A)$ with node set $\mathcal{W} = \{\psi_1, \ldots, \psi_n\}$ and edge set $\mathcal{E} = \{(\psi_i, \psi_j),\ i, j \text{ s.t. } A_{ji} \neq 0\}$. If $\psi_i \in \mathcal{W}$ is a root node, then the $i$:th row of $A$ is empty, i.e. $(A^T)[i] = 0$, and $e_i^T A = 0$. The root generates a zero eigenvalue and the elementary vector $e_i$ is an associated left eigenvector.

Also a dilation in the network generates one or several zero eigenvalues. The notion of dilation in a network was introduced together with structural controllability in Lin[26]. The in-neighbourhood set $T(\mathcal{S})$ of a set $\mathcal{S} \subset \mathcal{W}$ is the set of all nodes from which there exist an edge to a node in $\mathcal{S}$, i.e. $T(\mathcal{S}) = \{\psi_j | (\psi_j, \psi_i) \in \mathcal{E}, \psi_i \in \mathcal{S}\}$. $|\mathcal{S}|$

and $|T(\mathcal{S})|$ are the cardinality of set $\mathcal{S}$ and $T(\mathcal{S})$ respectively. The following definition of dilation is from Liu et al.[27]:

**Definition 1.** *The directed graph $\mathcal{G}(A)$ contains a dilation iff there is a subset $\mathcal{S}$ such that $|T(\mathcal{S})| < |\mathcal{S}|$.*

The root nodes are not allowed to belong to $\mathcal{S}$ but may belong to $T(\mathcal{S})$. If $\mathcal{G}(A)$ contains a dilation and the nodes are numbered such that $\mathcal{S} = \{\psi_{(n-|\mathcal{S}|+1)}, \ldots, \psi_n\}$, then the matrix $A$ has the form

$$A = \begin{bmatrix} P_1 \\ P_2, \end{bmatrix} \tag{14}$$

where the matrix $P_1$ has $(n-|\mathcal{S}|)$ rows and $n$ columns and $P_2$ is a $|\mathcal{S}|$-by-$n$ matrix with $|T(\mathcal{S})|$ non-zero columns. $P_2$ has no empty rows since there are no root nodes in $\mathcal{S}$. It holds that $\text{rank}(P_1) \leq n - |\mathcal{S}|$ and $\text{rank}(P_2) \leq |T(\mathcal{S})|$. Hence $\text{rank}(A) = \text{rank}(P_1) + \text{rank}(P_2) \leq n - |\mathcal{S}| + |T(\mathcal{S})| < n$. That is, a dilation in $\mathcal{G}(A)$ corresponds to a loss of rank in $A$, i.e., the dilation generates one or several zero eigenvalues. Furthermore, since $|T(\mathcal{S})| < |\mathcal{S}|$, the rows of $P_2$ are linearly dependent. This is equivalent to the existence of a vector $v = \begin{bmatrix} 0 & v_2^T \end{bmatrix}^T \in \mathbb{R}^n$ s.t. $v \neq 0$ and $v^T A = v_2^T P_2 = 0$. The vector $v$ is an eigenvector associated to the zero eigenvalue generated by the dilation. It has non-zero entries only at positions corresponding to the nodes in $\mathcal{S}$. The concept of dilation is exemplified in Figure S7(a).

In linear algebra, algorithms specialized in finding a sparse basis for the null space typically use graph methods to explore the roots and dilations in $\mathcal{G}(A)$ by means of the Dulmage-Mendelsohn decomposition and maximal matching. See Gilbert et al.[28] for a thorough review of both theory and algorithms. The term dilation is however not used in this litterature. In Definition 1, a dilation is defined based on the sets $\mathcal{S}$ and $T(\mathcal{S})$. The algorithms essentially try to partition $(\mathcal{S}, T(\mathcal{S}))$ into smaller separate pieces called *circuits*. By doing so, a sparse basis for the null space can be obtained. We will use "separate dilations" instead of circuits to give a more intuitive interpretation.

What is important in the context of unilateral controllability is that $\mathcal{N}_\ell(A)$ is the union of a set of subspaces related to the roots and separate dilations. For each of the subspaces, the vectors that span it have non-zero entries only on positions corresponding to the nodes that are "involved" in the dilation/root node. Fig. S7(b) illustrates

a network with a dilation that can be partitioned and Fig. S7(c) one that cannot.

### Positive spanning with structurally disjoint vectors

Assume that two vectors $a_i$ and $a_j \in \mathbb{R}^n$ are given. If $a_i[k] = 0$ for each $k$ s.t. $a_j[k] \neq 0$ and $a_j[l] = 0$ for each $l$ s.t. $a_i[l] \neq 0$ then we say that $a_i$ and $a_j$ are *structurally disjoint*. The positions of their non-zero entries are non-overlapping.

**Lemma 2.** *If the vectors $a_1, \ldots, a_r \in \mathbb{R}^n$ can be divided into two non-empty sets, $\mathcal{A}_1$ and $\mathcal{A}_2$ such that any $a_i \in \mathcal{A}_1$ and $a_j \in \mathcal{A}_2$ are structurally disjoint, then they can positively span $\mathbb{R}^n$ only if $r \geq n + 2$.*

*Proof.* From Theorem 1, if there is a strictly positive relation $\theta_1 a_1 + \cdots + \theta_r a_r = 0$, $\theta_i > 0$, then any vector $a_i$, $i = 1, \ldots, r$ is a linear combination of the other vectors, $a_i = \frac{-1}{\theta_i} \sum_{j \neq i} \theta_j a_j$.

We first show that $r = n+1$ is impossible. Assume that $a_1, \ldots, a_n$ linearly span $\mathbb{R}^n$. Then they must be linearly independent. If $a_{n+1} \in \mathcal{A}_1$ then the vectors in $\mathcal{A}_2$ are still linearly independent, and if $a_{n+1} \in \mathcal{A}_2$ then so are the vectors in $\mathcal{A}_1$. In any case there exists linearly independent vectors hence there cannot exist a strictly positive relation and $a_1, \ldots, a_r$ do not positively span $\mathbb{R}^n$. If on the other hand $a_1, \ldots, a_n$ does not linearly span $\mathbb{R}^n$, but $a_1, \ldots, a_{n+1}$ does, then instead $a_{n+1}$ must be linearly independent from $a_1, \ldots, a_n$ and the same argument holds. Finally, if $a_1, \ldots, a_{n+1}$ does not linearly span $\mathbb{R}^n$ then neither can they positively span $\mathbb{R}^n$.

When $r = n + 2$, take for instance $a_1, \ldots a_n$ linearly independent and $a_{n+1} = -\sum_{a_i \in \mathcal{A}_1} a_i$ and $a_{n+2} = -\sum_{a_j \in \mathcal{A}_2} a_j$. Then $\sum_{i=1,\ldots,r} a_i = 0$ is a strictly positive relation. The conditions of Theorem 1 are met and $a_1, \ldots a_r$ positively span $\mathbb{R}^n$. ■

By induction, this result generalizes to the case when $a_1, \ldots, a_r$ can be divided into arbitrarily many sets where the vectors of each set are structurally disjoint from the vectors of any other set.

When $V_0$ is constructed as in (4) from a sparse basis of $\mathcal{N}_\ell(A)$, then any two columns that originate from different subspaces are structurally disjoint. Also any two rows of $V_0$ which correspond to nodes in different dilations/roots are structurally disjoint. See Fig. 2 for an illustration and (17) for the special case of rooted

directed trees. When only control inputs that each act on a single node are considered, then the rows of $V_0$ become columns in $V_0^T B$ and the disjoint structure propagates. When $d$ is the number of structurally disjoint subspaces in $\mathcal{N}_\ell(A)$, the matrix $V_0^T B$ must have at least $\mu_0 + d$ columns for them to positively span $\mathbb{R}^{\mu_0}$. Since each column corresponds to one unilateral control input, the lower bound $N^r \geq \mu_0 + d$ follows.

# 6 Unilateral controls for some specific network structures

Results on $N^u$ and $N^r$ are given for a few specific networks in Table 1. The results for circular networks and rooted directed trees (RDT) are derived here. A single hub with outdegree is a special case of a RDT and is therefore not treated for itself.

### Circular network

The adjacency matrix of a circular network has the structure

$$A = \begin{bmatrix} 0 & \dots & 0 & a_{1n} \\ a_{21} & \ddots & & 0 \\ & \ddots & & \vdots \\ 0 & & a_{n,n-1} & 0 \end{bmatrix}. \tag{15}$$

The eigenvalues are the roots of

$$\lambda^n = a_{21} \cdot \ldots \cdot a_{n,n-1} \cdot a_{1n}.$$

All eigenvalues are simple and there are real eigenvalues only if the right hand side product is positive.

When there are no real eigenvalues, then the conditions for controllability with unilateral controls are the same as with unrestricted controls, i.e. controllability is achieved when the matrix $\begin{bmatrix} B & AB & A^2B & \dots & A^{n-1}B \end{bmatrix}$ has rank $n$. Because of the circular structure, this is achieved with a singe control input acting on any node.

If there are real eigenvalues, then two unilateral controls are needed. The selection of one positive and one negative control input

acting on the same node is a valid selection that is equivalent to one unrestricted control input. There can be several other selections of two unilateral control inputs that suffice. As an example, consider the cirular network with only positive edge-weights in Fig. 1(a) of the paper. It is shown to be unilaterally controllable with for instance $B = \begin{bmatrix} e_1 & -e_3 \end{bmatrix}$.

### Rooted directed trees

Given a rooted directed tree defined by the adjacency matrix $A \in \mathbb{R}^{n\times n}$, let $k_i$ be the outdegree of node $i$ and $f$ be the number of nodes with $k_i \geq 1$. Assume that the nodes are numbered such that $\psi_1$ is the root, $\psi_2, \ldots, \psi_f$ are the remaining nodes with out-neighbours, and $\psi_{f+1}, \ldots, \psi_n$ are the leaves. Introduce

$$\begin{aligned} &\gamma_0 = 1, \text{ and} \\ &\gamma_i = \gamma_{i-1} + k_i, \ i = 1, \ldots, f, \end{aligned}$$

i.e. the cumulative outdegree of the $i$ first nodes plus 1. Note that $\gamma_f = n$. The adjacency matrix for a general RDT is

$$A = \begin{bmatrix} & & & & \\ a_{(\gamma_0+1,1)} & & & & \\ \vdots & & & & \\ a_{(\gamma_1,1)} & & & & \\ & \ddots & & & \\ & & a_{(\gamma_{f-1}+1,f)} & & \\ & & \vdots & & \\ & & a_{(\gamma_f,f)} & & \end{bmatrix}. \tag{16}$$

In (16), blank spaces are zeros and the columns $A[f+1], \ldots, A[n]$ are empty. The out-neighbours of $\psi_i,\ i \in 1, \ldots, f$ are $\psi_{\gamma_{i-1}+1}, \ldots, \psi_{\gamma_i}$. From (16) we have that $\text{rank}(A) = f$, hence $\mu_0 = n - f = n_{\text{leaf}}$ for a RDT. Since $\lambda_0 = 0$ is the only eigenvalue only $V_0^T B$ must be considered when evaluating controllability and condition *ii)* of Theorem 4. Given $A$ as in (16), it is straightforward to verify that the columns of the matrix $V_0$ below form a sparse basis for $\mathcal{N}_\ell(A)$.

$$V_0 = \begin{bmatrix} 1 & & & & \\ & \begin{matrix} a_{(\gamma_1,1)} & & 0 \\ & \ddots & \\ 0 & & a_{(\gamma_1,1)} \\ -a_{(\gamma_0+1,1)} & \cdots & -a_{(\gamma_1-1,1)} \end{matrix} & & & \\ & & \ddots & & \\ & & \ddots & & \\ & & & & \begin{matrix} a_{(\gamma_f,f)} & & 0 \\ & \ddots & \\ 0 & & a_{(\gamma_f,f)} \\ -a_{(\gamma_{f-1}+1,f)} & \cdots & -a_{(\gamma_f-1,f)} \end{matrix} \end{bmatrix}. \tag{17}$$

Besides the first entry, the diagonal blocks are of size $k_i \times (k_i - 1)$, $i = 1, \dots f$. The first column spans the subspace of $\mathcal{N}_\ell(A)$ that is generated by the root and the columns of the following diagonal blocks span the subspaces that are generated by nodes with outgoing edges. Notice that a node with outdegree $k_i = 1$ adds a 1-by-0 block to $V_0$, i.e. an empty row. Only nodes with $k_i \geq 2$ add to the dimension of $\mathcal{N}_\ell(A)$. Define the set $\mathcal{I} = \{i \in 1, \dots, n \text{ s.t. } k_i \geq 2\}$. Each node $\psi_i$, $i \in \mathcal{I}$, constitutes a separate dilation with its children defined by the sets $\mathcal{S}_i = \{\psi_{\gamma_{i-1}+1}, \dots, \psi_{\gamma_i}\}$ and $T(\mathcal{S}_i) = \{\psi_i\}$. Hence, $d = |\mathcal{I}| + 1$ (also accounting for the root).

The following set of control inputs guarantee unilateral controllability:

$$\begin{aligned}\mathcal{B} =& \{e_1, -e_1\} \cup \\ & \{-\text{sign}(a_{(\gamma_{i-1}+j,i)}/a_{(\gamma_i,i)})e_{(\gamma_{i-1}+j)} | \forall i \in \mathcal{I},\ j = 1, \dots, k_i\},\end{aligned}$$

and the matrix $B$ is constructed by stacking the vectors of $\mathcal{B}$. Notice that the root node and all nodes with a sibling, i.e. nodes that share in-neighbour with some other node, are targeted with this selection of control inputs. Controllability can be verified using Theorem 4: The perhaps easiest way to verify the rank condition *i)* is to use the equivalent PBH test. It requires the matrix $[sI - A\ B]$ to have full row rank for every complex scalar $s$. Since $\lambda = 0$ is the only eigenvalue of $A$ it is however enough to verify this condition for $s = 0$: $\text{rank}([-A\ B]) = n$. Notice in (16) that only rows of $A$ that correspond to the root and to siblings are linearly dependent. But since all these nodes are targeted by control inputs, the corresponding

rows of $[-A\ B]$ are linearly independent, hence $[-A\ B]$ has full row rank ($= n$). The condition *ii)* is met if there is a strictly positive relation between the columns of $V^T B$. A strictly positive relation can be constructed with the ratios $|a_{(\gamma_{i-1}+j,i)}/a_{(\gamma_i,i)}|$, $i \in \mathcal{I}, j = 1, \ldots, k_i$ as coefficients in (1). The number of unilateral control inputs is $N^r = |\mathcal{B}| = 2 + \sum_{i\in\mathcal{I}} k_i$. Using $\mu_0 = 1 + \sum_{i\in\mathcal{I}}(k_i - 1)$, we obtain $|\mathcal{B}| = \mu_0 + d$. Hence, the topology induced lower bound is obtained and the number of unilateral controls is minimal. Alternatively, we can write $N^r = n_{\text{leaf}} + |\mathcal{I}| + 1$, where it is used that $n_{\text{leaf}} = \mu_0$ for RDTs. The number $N^u$ is easily obtained using for instance the *structural controllability* framework. The conditions for controllability are then formulated in terms of *inaccessible nodes* and *dilations*, see for instance Commault[24]. Straight forward application of such methods gives $N^u = \mu_0$. This is obtained for instance by choosing as driver nodes the root node and for each node with $k_i \geq 2$ all but one of its children. (Then there are no inaccessible nodes or dilations.)

## References


[1] Milo, R. *et al.* Network motifs: simple building blocks of complex networks. *Science* **298**, 824–827 (2002).

[2] Oda, K., Matsuoka, Y., Funahashi, A. & Kitano, H. A comprehensive pathway map of epidermal growth factor receptor signaling. *Molecular systems biology* **1** (2005).

[3] Oda, K. & Kitano, H. A comprehensive map of the toll-like receptor signaling network. *Molecular systems biology* **2** (2006).

[4] Oda, K. *et al.* Molecular interaction map of a macrophage. *AfCS Research Reports* **2**, 1–12 (2004).

[5] Förster, J., Famili, I., Fu, P., Palsson, B. Ø. & Nielsen, J. Genome-scale reconstruction of the saccharomyces cerevisiae metabolic network. *Genome research* **13**, 244–253 (2003).

[6] Reed, J. L., Vo, T. D., Schilling, C. H. & Palsson, B. O. An expanded genome-scale model of escherichia coli k-12 (i jr904 gsm/gpr). *Genome biology* **4**, 1 (2003).

[7] Watts, D. J. & Strogatz, S. H. Collective dynamics of 'small-world'networks. *nature* **393**, 440–442 (1998).

[8] Menck, P. J., Heitzig, J., Kurths, J. & Schellnhuber, H. J. How dead ends undermine power grid stability. *Nature communications* **5** (2014).

[9] Josz, C., Fliscounakis, S., Maeght, J. & Panciatici, P. Ac power flow data in matpower and qcqp format: itesla, rte snapshots, and pegase. *arXiv preprint arXiv:1603.01533* (2016).

[10] Ripeanu, M., Foster, I. & Iamnitchi, A. Mapping the gnutella network: Properties of large-scale peer-to-peer systems and implications for system design. *arXiv preprint cs/0209028* (2002).

[11] Karrer, B., Newman, M. E. & Zdeborová, L. Percolation on sparse networks. *Physical review letters* **113**, 208702 (2014).

[12] Leskovec, J. & Krevl, A. SNAP Datasets: Stanford large network dataset collection. `http://snap.stanford.edu/data` (2014).

[13] Madenjian, C. P. *et al.* Dynamics of the lake michigan food web, 1970-2000. *Canadian Journal of Fisheries and Aquatic Sciences* **59**, 736–753 (2002).

[14] Ulanowicz, R. E. & DeAngelis, D. L. Network analysis of trophic dynamics in south florida ecosystems. *US Geological Survey Program on the South Florida Ecosystem* **114** (2005).

[15] Batagelj, V. & Mrvar, A. Pajek data sets. `http://pajek.imfm.si/doku.php?id=data:index`. Accessed: 2016-09-21.

[16] Hidalgo, C. A. & Hausmann, R. The building blocks of economic complexity. *proceedings of the national academy of sciences* **106**, 10570–10575 (2009).

[17] De Domenico, M., Nicosia, V., Arenas, A. & Latora, V. Structural reducibility of multilayer networks. *Nature communications* **6** (2015).

[18] Yazdani, A. & Jeffrey, P. Complex network analysis of water distribution systems. *Chaos: An Interdisciplinary Journal of Nonlinear Science* **21**, 016111 (2011).

[19] Davis, C. Theory of positive linear dependence. *American Journal of Mathematics* **76**, pp. 733–746 (1954).

[20] Wright, S. E. A note on positively spanning sets. *The American Mathematical Monthly* **107**, 364–366 (2000).

[21] Brammer, R. F. Controllability in linear autonomous systems with positive controllers. *SIAM Journal on Control* **10**, 339–353 (1972).

[22] Heymann, M. & Stern, R. J. Controllability of linear systems with positive controls: Geometric considerations. *Journal of Mathematical Analysis and Applications* **52**, 36–41 (1975).

[23] Alon, N., Moshkovitz, D. & Safra, S. Algorithmic construction of sets for k-restrictions. *ACM Transactions on Algorithms (TALG)* **2**, 153–177 (2006).

[24] Commault, C. & Dion, J.-M. Input addition and leader selection for the controllability of graph-based systems. *Automatica* **49**, 3322 – 3328 (2013).

[25] Yuan, Z., Zhao, C., Di, Z., Wang, W.-X. & Lai, Y.-C. Exact controllability of complex networks. *Nat Commun* **4** (2013).

[26] Lin, C. T. Structural controllability. *Automatic Control, IEEE Transactions on* **19**, 201–208 (1974).

[27] Liu, Y.-Y., Slotine, J.-J. & Barabási, A.-L. Controllability of complex networks. *Nature* **473**, 167–173 (2011).

[28] Gilbert, J. R. & Heath, M. T. Computing a sparse basis for the null space. *SIAM Journal on Algebraic Discrete Methods* **8**, 446–459 (1987).

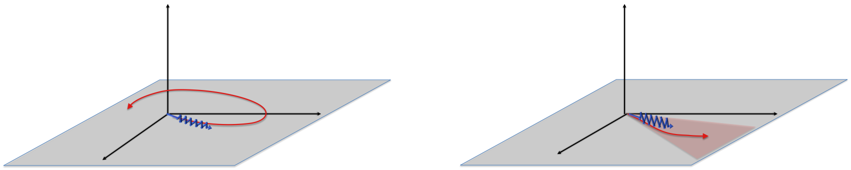

Complex conjugate eigenvalues
(a)

Real eigenvalue
(b)

Figure S1: (a): When the eigenspace of a pair of complex conjugate eigenvalues is excited by a unilateral control, then the imaginary part of the eigenvalue "rotates" the state vector, hence the reachable set is not confined to a half space. (b): When the eigenspace of a real eigenvalue is excited by a positive control, instead, the reachable set has the structure of a cone (red shaded area), in which case controllability is not achieved.

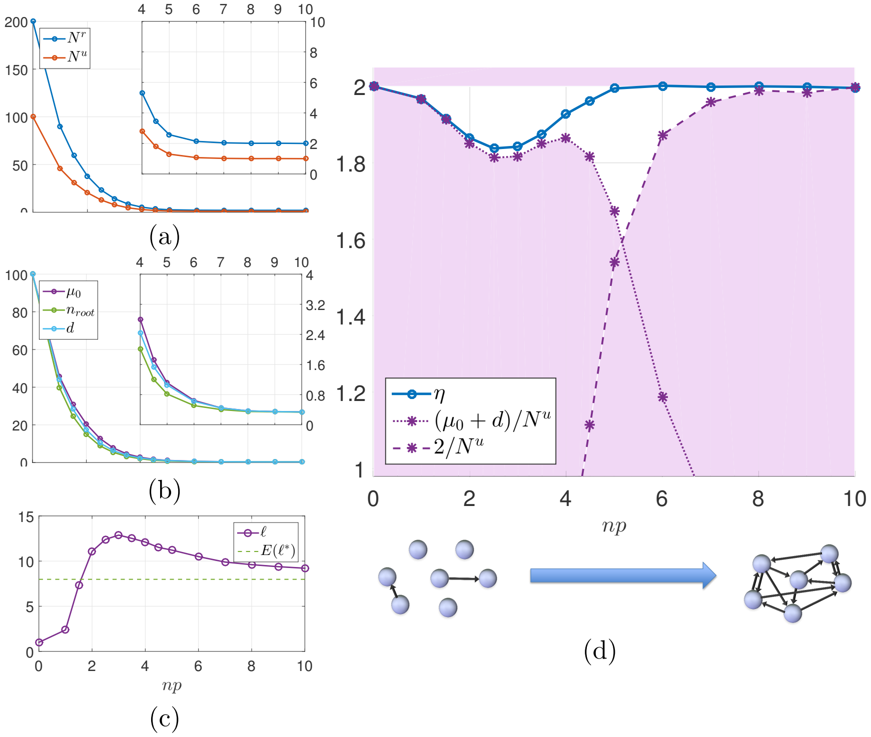


Figure S2: Erdős–Rényi networks with $n = 100$ generated with different edge-probabilities, $p$. Note that the x-axis shows $np$ which is the expected indegree/outdegree for the nodes. For each configuration, 1000 random networks are generated with edge weights sampled from a normal distribution. Averaged values are shown. The results are well in line with the corresponding results for larger networks ($n = 1000$) in Fig. 3 of the paper. The size of the networks thus appears to have no real importance for the analysis of controllability with unilateral control inputs.

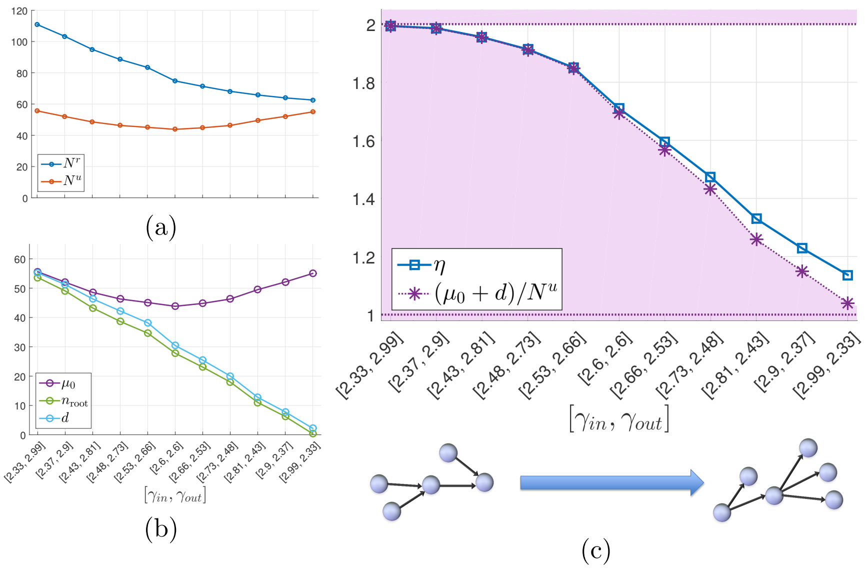


Figure S3: Scale-free networks with $n = 100$ are generated with different indegree and outdegree exponents, $\gamma_{\text{in}}$ and $\gamma_{\text{out}}$. For each configuration of $\gamma_{\text{in}}$ and $\gamma_{\text{out}}$, 1000 random networks are generated and the figures show their averaged values. Edge weights are sampled from a normal distribution. The total number of edges is the same for all configurations. The results are well in line with the corresponding results for larger networks ($n = 1000$) in Fig. 4 of the paper.

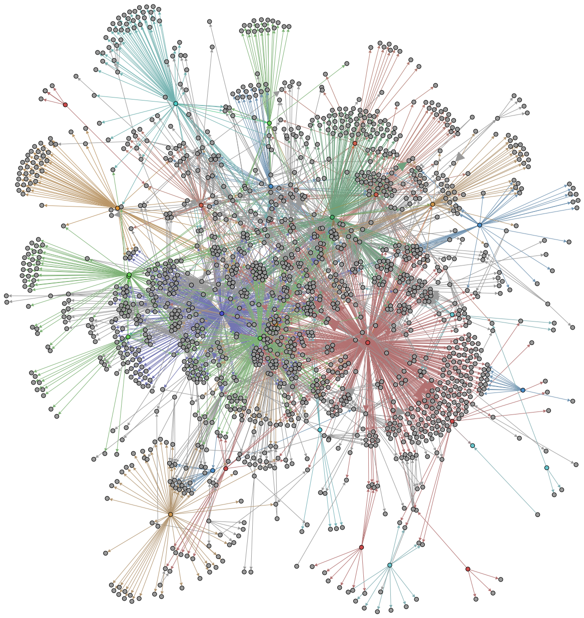

(a)

| Network statistics | |
|---|---|
| Nodes | 1623 |
| Edges | 2620 |
| Edge density | 0.14% |
| $h_{\max}$ | 11 |
| $\mu_0$ | 1466 |
| $n_{\text{leaf}}$ | 1466 |
| $n_{\text{root}}$ | 29 |
| $d$ | 32 |
| $N^r$ | 1692 |
| $N^u$ | 1466 |
| $\eta$ | 1.16 |

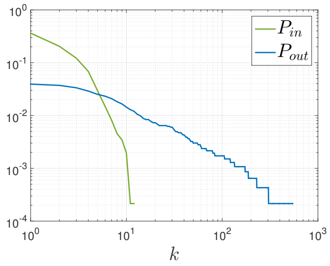


(b)

Figure S4: E.coli gene regulatory network. (a): The network has a DAG-like structure with only a few layers. It has a number of hubs with high outdegree and many leaves, but it has no hubs with high indegree and only few roots. (b): The indegree and outdegree distributions are similar to those of a directed scale-free network with $\gamma_{\text{in}} > \gamma_{\text{out}}$. A large number of controls are required to achieve controllability but the ratio $\eta$ is low.

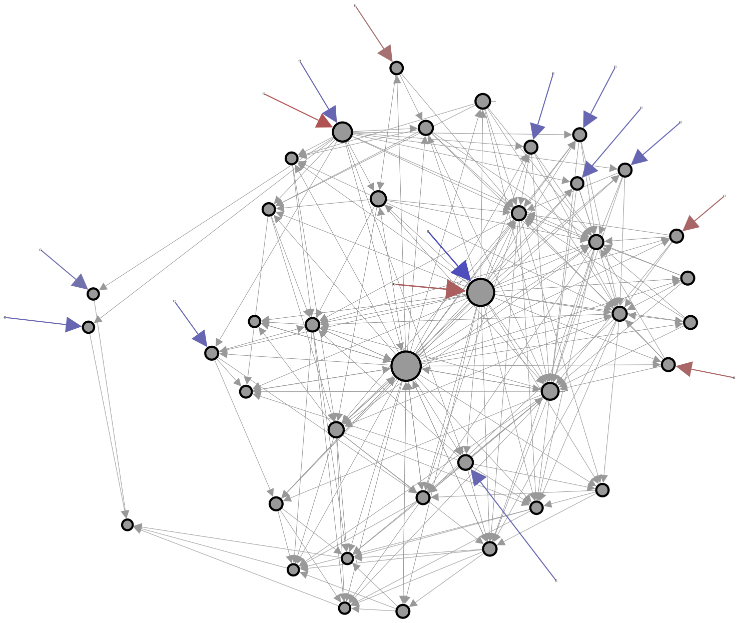

(a)

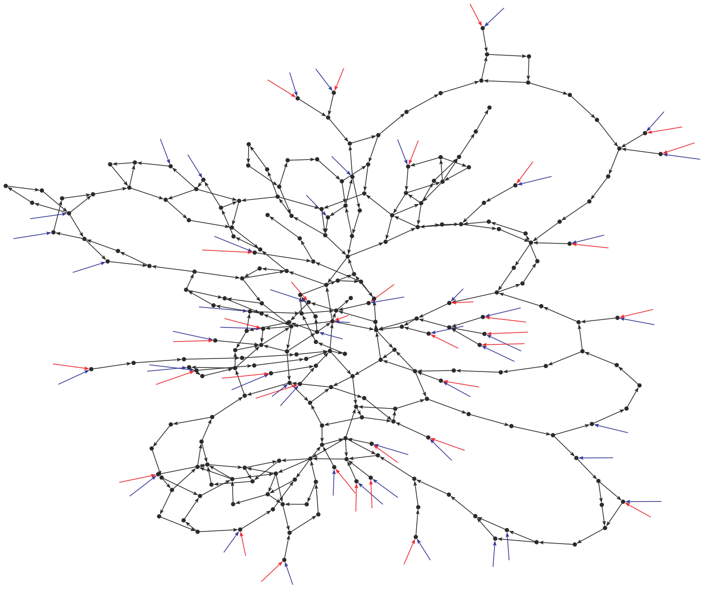

(b)

Figure S5: Examples of networks with their unilateral controls guaranteeing controllability. (a): Lake Michigan food web[13]. The graph represents a dynamical model in which each state represents the population of a species. A minimal set of positive (blue) and negative (red) unilateral control inputs that make the network controllable is shown. For instance feeding and breeding, hunting and fishing are naturally unilateral control inputs in an ecosystem. (b): Graph of the Northern European power grid. The node and edge-lists are retrieved from Menck et al.[8].

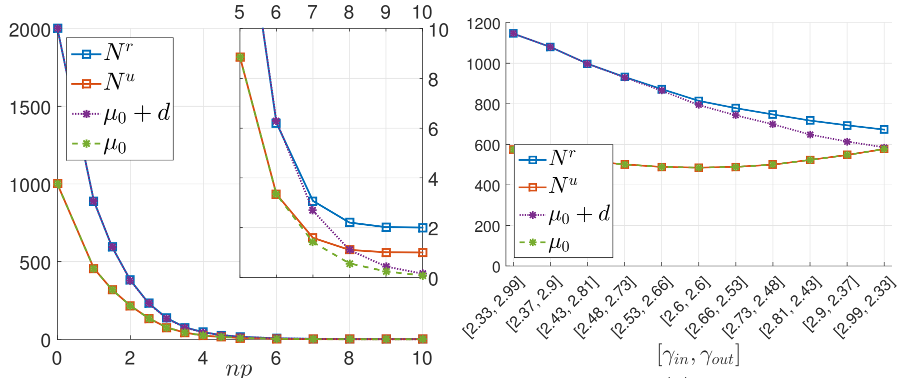


(a) (b)

Figure S6: (a): Here we can see how closely $N^r$ and $N^u$ follow their respective lower bounds $\mu_0 + d$ and $\mu_0$ for the Erdős–Rényi networks with $n = 1000$ shown in Fig. 3 of the paper. There is a significant difference only for dense networks with high expected indegree and outdegree ($np$). (b): The same comparison, but for directed scale-free networks (Fig. 4). In this case $N^u$ meets its lower bound $\mu_0$ for all configurations. Also $N^r$ follows its lower bound well on the left side of the plot, but the bound is not achieved for the networks in the right end. These are networks with hubs having significantly higher outdegree than indegree.

(a)

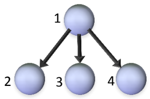


$\mathcal{S} = \{\psi_2, \psi_3, \psi_4\}$, $T(\mathcal{S}) = \{\psi_1\}$

$$A = \begin{bmatrix} 0 & 0 & 0 & 0 \\ 1 & 0 & 0 & 0 \\ 1 & 0 & 0 & 0 \\ 1 & 0 & 0 & 0 \end{bmatrix}, \; v_{0,1} = \begin{bmatrix} 0 \\ -1 \\ 1 \\ 0 \end{bmatrix}, \; v_{0,2} = \begin{bmatrix} 0 \\ -1 \\ 0 \\ 1 \end{bmatrix}, \; v_{0,3} = \begin{bmatrix} 1 \\ 0 \\ 0 \\ 0 \end{bmatrix}$$

(b)

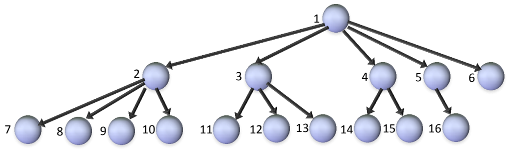


$\mathcal{S}_1 = \{\psi_2, \psi_3, \psi_4, \psi_5, \psi_6\}$, $T(\mathcal{S}_1) = \{\psi_1\}$,
$\mathcal{S}_2 = \{\psi_7, \psi_8, \psi_9, \psi_{10}\}$, $T(\mathcal{S}_2) = \{\psi_2\}$,
$\mathcal{S}_3 = \{\psi_{11}, \psi_{12}, \psi_{13}\}$, $T(\mathcal{S}_3) = \{\psi_3\}$,
$\mathcal{S}_4 = \{\psi_{14}, \psi_{15}\}$, $T(\mathcal{S}_4) = \{\psi_4\}$.

(c)

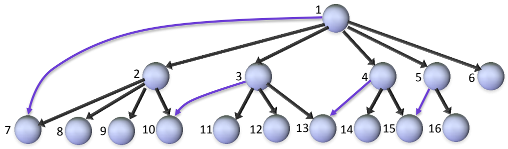


$\mathcal{S} = \{\psi_2, \ldots, \psi_{16}\}$,
$T(\mathcal{S}) = \{\psi_1, \psi_2, \psi_3, \psi_4, \psi_5\}$.

Figure S7: (a): A hub with outdegree three exemplifies the concept of dilation. Highlighted rows in $A$ correspond to $P_2$ in (14). $P_2$ has $|T(\mathcal{S})| = 1$ non-zero columns and $|\mathcal{S}| - |T(\mathcal{S})| = 2$ linearly independent eigenvectors of $\lambda = 0$ can be associated with the dilation ($v_{0,1}$ and $v_{0,2}$). The last eigenvector ($v_{0,3}$) comes from the root node. The vectors $v_{0,1}$, $v_{0,2}$ and $v_{0,3}$ form a basis for $\mathcal{N}_\ell(A)$. (b): The RDT studied in Fig. 2(a) of the paper has four separate dilations, one for each node with outdegree $\geq 2$. The sets $\mathcal{S}_1, \ldots, \mathcal{S}_4$ are disjoint and so are also $T(\mathcal{S}_1), \ldots, T(\mathcal{S}_4)$. Furthermore, $\mathcal{S}_i,\ i = 1, \ldots 4$, contains all the children of $T(\mathcal{S}_i)$. (c): The dilation in the DAG studied in Fig. 2(c) of the paper cannot be partitioned.
